# Psychotic experiences and negative symptoms from adolescence to emerging adulthood: developmental trajectories and associations with polygenic scores and childhood characteristics

**DOI:** 10.1017/S0033291722002914

**Published:** 2023-09

**Authors:** Laura Havers, Sophie von Stumm, Alastair G. Cardno, Daniel Freeman, Angelica Ronald

**Affiliations:** 1 Department of Psychological Sciences, Birkbeck, University of London, London, UK; 2 Department of Education, University of York, Heslington, UK; 3 Division of Psychological and Social Medicine, University of Leeds, Leeds, UK; 4 Department of Psychiatry, University of Oxford, Oxford, UK; 5 Oxford Health NHS Foundation Trust, Oxford, UK

**Keywords:** Adolescence, community, developmental trajectories, emerging adulthood, growth mixture modelling, negative symptoms, polygenic scores, psychotic experiences

## Abstract

**Background:**

Psychotic experiences and negative symptoms (PENS) are common in non-clinical populations. PENS are associated with adverse outcomes, particularly when they persist. Little is known about the trajectories of PENS dimensions in young people, nor about the precursory factors associated with these trajectories.

**Methods:**

We conducted growth mixture modelling of paranoia, hallucinations, and negative symptoms across ages 16, 17, and 22 in a community sample (*N =* 12 049–12 652). We then described the emergent trajectory classes through their associations with genome-wide polygenic scores (GPS) for psychiatric and educational phenotypes, and earlier childhood characteristics.

**Results:**

Three trajectory classes emerged for paranoia, two for hallucinations, and two for negative symptoms. Across PENS, GPS for clinical help-seeking, major depressive disorder, and attention deficit hyperactivity disorder were associated with increased odds of being in the most elevated trajectory class (OR 1.07–1.23). Lower education GPS was associated with the most elevated trajectory class for hallucinations and negative symptoms (OR 0.77–0.91). Conversely for paranoia, higher education GPS was associated with the most elevated trajectory class (OR 1.25). Trajectory class associations were not significant for schizophrenia, obsessive-compulsive disorder, bipolar disorder, or anorexia GPS. Emotional/behaviour problems and life events in childhood were associated with increased odds of being in the most elevated trajectory class across PENS.

**Conclusions:**

Our results suggest latent heterogeneity in the development of paranoia, hallucinations, and negative symptoms in young people that is associated with specific polygenic scores and childhood characteristics.

## Introduction

Experiences that are characteristic of psychosis are frequently observed in community samples (Healy et al., 2019; McGrath et al., 2015). Psychotic experiences such as delusions, paranoia and hallucinations, and negative symptoms, including flat affect and poverty of speech, have been reported in childhood and adolescence, and across the lifespan (Barragan, Laurens, Navarro, & Obiols, 2011; Dhossche, Ferdinand, Ende, Hofstra, & Verhulst, 2002; Dominguez, Saka, Lieb, Wittchen, & van Os, 2010; Kelleher et al., 2012; Maric, Krabbendam, Vollebergh, de Graaf, & van Os, 2003; Ronald et al., 2014). Whilst psychotic experiences reported by individuals in community samples generally abate (Linscott & van Os, 2013; van Os, Linscott, Myin-Germeys, Delespaul, & Krabbendam, 2009), persistence compared to the transience of psychotic experiences and negative symptoms (PENS) has been found to predict poor clinical and functional outcomes (e.g. Hielscher *et al*. 2021; Kaymaz *et al*. 2012; Mackie, Castellanos-Ryan, & Conrod, 2011; Wigman *et al*. 2011b). For example, psychotic experiences reported in an adolescent community sample were associated with increased odds for psychotic impairment in a dose–response manner, with odds ratios of 1.5, 5, and 9.9 for the presence of psychotic experiences at one, two, and three time-points, respectively (Dominguez, Wichers, Lieb, Wittchen, & van Os, 2011). Less is known about the developmental course of negative symptoms in the community, but findings from a small number of studies suggest that persistence is associated with adverse outcomes including psychotic and functional impairment (Dominguez et al., 2010; Janssens et al., 2016).

Previous longitudinal studies have often focussed on total scores or aggregated measures of psychotic experiences. Persistence of these experiences has been found to be associated with family background characteristics such as familial psychiatric history and socioeconomic status (SES); and with characteristics reported in childhood such as lower educational attainment, childhood trauma and other adverse life events, and emotional and behavioural problems (Bourque, Afzali, O'Leary-Barrett, & Conrod, 2017; Cougnard et al., 2007; DeVylder, Lehmann, & Chen, 2015; Janssens et al., 2016; Kalman, Bresnahan, Schulze, & Susser, 2019; Rammos et al., 2021).

However, PENS that are reported in the community have a multidimensional psychometric structure (Ronald et al., 2014; Stefanis et al., 2004; Yung et al., 2009), and specific dimensions show distinct associations with other types of psychopathology (Armando et al., 2010; Ronald et al., 2014; Wigman et al., 2011c; Yung et al., 2009), environmental exposures (Cosgrave et al., 2021; Shakoor et al., 2015), and genetic factors (Zavos et al., 2014). Gaining a better understanding of both the development of *separate* PENS dimensions, and the precursory factors associated with their development is important. It will allow for the delineation of dimension-specific theoretical models, which may be implemented to identify and help individuals at risk for both concurrent psychopathology and later poor outcomes (Armando et al., 2010; Cosgrave et al., 2021; Steenkamp et al., 2021; Yung et al., 2009).

The handful of studies that have investigated the development of paranoia/delusions and hallucinations separately have mainly relied on measures of a few items (Connell et al., 2016; De Loore et al., 2011; Hielscher et al., 2021; Sheaves et al., 2016; Steenkamp et al., 2021). These studies found the persistence of paranoia/delusions and hallucinations to be associated with a range of maladies. Building on the findings of these studies that used brief measurement tools, measures that assess quantitative variation across a broader range of experiences (e.g. Bartels-Velthuis, van de Willige, Jenner, van Os, & Wiersma, 2011; Freeman *et al*. 2012) have the potential to further enhance understanding of PENS by capturing experiences that may otherwise go undetected (Mitchell et al., 2017).

In terms of genetic factors, several studies have investigated their influence on PENS at single time-points or assessments (Ronald & Pain, 2018), and findings from a small number of family studies further suggest that genetic factors are associated with the development of PENS (Ericson, Tuvblad, Raine, Young-Wolff, & Baker, 2011; Havers, Taylor, & Ronald, 2019; Janssens et al., 2016; Wigman et al., 2011a). The prior study with the largest sample size (*N* = 1448 twin pairs) found that 38–62% of the covariance in separate PENS dimensions measured across two time-points in adolescence was accounted for by genetic influences (Havers et al., 2019). Genome-wide polygenic scores (GPS) can also be used as an index of an individual's polygenic propensity to a given outcome. Only one study to date has employed polygenic score methods in the context of the persistence of PENS. This study reported a null association between schizophrenia GPS and the persistence of aggregated psychotic experiences, measured across ages 12–24 in the Avon Longitudinal Study of Parents and Children (Rammos et al., 2021). PENS likely reflect vulnerability for poor functional and clinical outcomes, broadly, rather than solely for psychosis (Healy et al., 2019; van Os & Reininghaus, 2016; Yung et al., 2009): the extent to which the development of PENS dimensions is associated with polygenic liability across a broad range of phenotypes, including psychiatric disorders, clinical help-seeking, intelligence and educational attainment, is therefore of interest, but has not previously been tested.

Previous studies that have investigated trajectories of PENS have done so primarily by manually grouping individuals according to the presence or absence of these experiences across time-points (e.g. Cougnard *et al*. 2007; Dominguez *et al*. 2011; Hafeez & Yung, 2021; Hielscher *et al*. 2021; Janssens *et al*. 2016; Rammos *et al*. 2021; Steenkamp *et al*. 2021; van Rossum, Dominguez, Lieb, Wittchen, & van Os, 2011). In contrast, latent variable modelling [specifically here, growth mixture modelling (GMM)] can be used, figuratively, to investigate whether individuals can be classified according to similarities in their underlying, latent trajectories (Herle et al., 2020). Of the studies that have used GMM, multiple latent trajectory classes including a persistent or increasing class have been identified for broadly-defined psychotic experiences measured across adolescence (Bourque et al., 2017; Lin et al., 2011; Mackie et al., 2011, 2013; Thapar et al., 2012; Wigman et al., 2011c), and adulthood (Wigman et al., 2011a). Yet, there are currently no published findings that estimate the latent development of paranoia and hallucinations separately, or of negative symptoms, reported in the community.

The current study builds on prior research by modelling latent heterogeneity in the development of separate PENS dimensions. We focused on the period of mid-late adolescence to emerging adulthood, which is a common period of onset for a range of mental health problems, including psychosis (Kessler et al., 2007; Kim-Cohen et al., 2003; Maibing et al., 2015). As pre-registered (https://osf.io/pax6k), we hypothesised that multiple classes, including a persistent class, would be identified for each of paranoia, hallucinations, and negative symptoms, and that persistence would be associated with the following: (i) lower SES and family psychiatric history, (ii) more emotional and behavioural difficulties, more life events, and lower educational attainment (both in childhood and adulthood), and (iii) higher GPS of psychiatric and clinical help-seeking outcomes, and lower GPS of intelligence and educational attainment. We also predicted that male sex would be associated with persistent negative symptoms (e.g. Dominguez *et al*. 2010; Roy, Maziade, Labbé, & Mérette, 2001).

## Methods

### Participants

Participants were part of the Twins Early Development Study (TEDS). Families of twins born 1994–1996 in England and Wales were invited by the Office for National Statistics to take part on behalf of TEDS. Sixteen thousand eight hundred and ten (16 810) families responded to the invitation. Online Supplementary Table S1 shows participation rates and exclusion details (see Rimfeld et al., 2019 for a recent overview of TEDS). Paranoia and hallucinations (as well as the additional measures at age 22) were self-reported, and negative symptoms were reported by parents (*N* *=* 12 049–12 652) at mean ages 16.32 (s.d. 0.69), 17.06 (s.d. 0.88), and 22.85 (s.d. 0.88) (online Supplementary Table S2). Online Supplementary Tables S4–S6 show demographic comparisons between individuals with complete and incomplete PENS data. Earlier in the study, parents completed assessments of their children's behaviour at mean age 7.06 (s.d. 0.25), and teachers reported educational achievement at mean age 7.20 (s.d. 0.27).

### Measures

#### PENS

Paranoia, hallucinations, and negative symptoms were assessed using subscales of the Specific Psychotic Experiences Questionnaire (SPEQ; Ronald et al., 2014), described in online Supplementary Information S1. *Paranoia* was measured by 15 items adapted from the Paranoia Checklist (Freeman et al., 2005), and *hallucinations* by nine items adapted from the Cardiff Anomalous Perceptions Scale (Bell, Halligan, & Ellis, 2006), both on a 6-point scale and both adapted for use in adolescents by clinical experts (Ronald et al., 2014). *Negative symptoms* were measured by eight items on a 4-point scale, adapted from the Scale for the Assessment of Negative Symptoms (Andreasen, 1982). Descriptive statistics are reported in online Supplementary Table S2. Reliability and validity information regarding use of the SPEQ in the current sample is reported in Ronald et al. (2014).

#### Additional measures

At ages 7 and 22, the Strengths and Difficulties Questionnaire (SDQ; Goodman, 1997) was used to assess emotional and behavioural problems, and the Short Mood and Feeling Questionnaire (MFQ; Angold, Costello, Messer, and Pickles, 1995) was used at age 22 to assess depressive symptoms. The assessment measures for SES, family psychiatric history, educational attainment, and life events are described in online Supplementary Information S2.

#### GPS

Genotyping of participants is described in online Supplementary Information S3. GPSs were calculated by other TEDS researchers (Selzam et al., 2018, 2019). Descriptions of the GPSs and their calculation are detailed in online Supplementary Information S4. Standardized residuals of the GPS regressed on the first 10 principal components of ancestry, batch, and chip were used. GPS corresponding to the most predictive fraction (*f*) of causal markers were used in the association analyses (online Supplementary Information S4 and Tables S14, S24, S34).

### Statistical analyses

A structural equation modelling framework was used for all analyses, using Mplus (version 8.6). Observed total score data at each age was modelled using full information maximum likelihood to accommodate missing data under the assumption that data were missing at random, and robust estimation was used to accommodate multivariate nonnormality of residuals. Data from related individuals were accommodated by adjustment to the standard errors, with the family ID specified as the unit of clustering.

Prior to conducting GMM, longitudinal measurement invariance was assessed, and a series of latent growth curve models (LGCM) were run to determine the optimal functional form of growth. GMM was used to identify latent trajectory classes using the growth form suggested by the LGCM. GMM extends LGCM by parameterising the trajectories of a prespecified number of heterogenous latent classes (online Supplementary Fig. S1) (Ram & Grimm, 2009).

A 1-class model was first estimated, followed by an increasing number of classes, up to the point where there was consistent nonconvergence (Jung & Wickrama, 2008). Within each *k*-class (where *k* refers to the number of classes), two models were initially run: A latent class growth analysis model (Model LCGA, with no variance on the latent growth factors), and an unconstrained GMM (Model 0). The following parameters were freely estimated in Model 0: (i) growth factor means, (ii) growth factor variances and covariances, (iii) residual variances. For *k* > 1 models, these parameters were estimated for each class. A series of constrained models were run where there were convergence issues with these models (Sijbrandij et al., 2020; van de Schoot, Sijbrandij, Winter, Depaoli, & Vermunt, 2017).

For the subsequently tested models, variance parameter constraints were as follows: Model 1A: Within-class residual variances. Model 1B: Between-class residual variances. Model 1C: Between-class growth factor variances. Model 2A: Within-class and between-class residual variances. Model 2B: Within-class residual variances and between-class growth factor variances. Model 2C: Between-class residual variances and between-class growth factor variances.

Online Supplementary Fig. S2 shows a decision-making flowchart regarding estimation specifications and model adjustments. Variation in time-scores (ages) was modelled by allowing individual (random) slope factor loadings (Mehta & West, 2000), specified using TSCORES in Mplus.

For each *k-*class, the model with the lowest BIC value was selected. These models were rerun using the two seed values corresponding to the highest replicated loglikelihood value (Jung & Wickrama, 2008; Shireman, Steinley, & Brusco, 2016). Where BIC values were indistinguishable (difference <10), the AIC was referred to and the model with the lowest AIC value (difference >2) was selected. The best fitting overall model was determined by jointly considering, (i) BIC (and AIC where necessary), (ii) entropy values (with a value of one reflecting perfect classification accuracy), (iii) empirical and theoretical plausibility of the within-class parameter estimates and overall solution. Two sets of post-hoc sensitivity tests were conducted, described in online Supplementary Information S5. Likelihood ratio tests for comparing *k-*class with *k-*1-class GMMs are not available for models with individual time-scores.

Multinomial logistic regression analyses were conducted to assess the relationship between the family background, age 7, and GPS variables (specified as auxiliary variables), and the latent class variable, using the automatically implemented ‘3-step’ procedure in Mplus (Asparouhov & Muthén, 2014; Vermunt, 2017). The automatically implemented ‘BCH’ procedure was used to estimate the class-specific means of all auxiliary variables (Asparouhov & Muthén, 2014; Bakk & Vermunt, 2016; Bolck, Croon, & Hagenaars, 2004): Both methods estimate the GMM and the most likely class membership values, and then make estimates of the relations between the auxiliary variables and the latent class variable, adjusted for classification error. The False Discovery Rate method was used to correct for multiple testing at *q* < 0.05 (Benjamini & Hochberg, 1995). Single-predictor regressions were first run. Significant predictors (at *q* < 0.05) were entered into multiple-predictor regressions.

## Results

### Descriptive statistics

Descriptive statistics for PENS at each age are shown in online Supplementary Table S2. Cross-age correlation coefficients are shown in online Supplementary Table S3.

### Measurement invariance

Partial scalar-level invariance was found for paranoia and hallucinations, and partial strict-level invariance was found for negative symptoms (online Supplementary Tables S7, S18, S28).

### LGCM

Linear growth models provided an acceptable approximation of the data across PENS (online Supplementary Tables S8, S19, S29; parameter estimates shown in online Supplementary Tables S9, S20, S30). Quadratic and latent basis models provided a better fit than the linear model for hallucinations, though these models were highly constrained (to achieve just- and over-identification). Linear models were taken forward in the interest of parsimony and consistency, to reduce the risk of overfitting, and for simpler estimation using individual time-scores (Sterba, 2014). Online Supplementary Figs S3–S5 show spaghetti plots for observed individual trajectories and mean trajectories estimated in the LGCMs.

### GMM

**Fig. 1.**
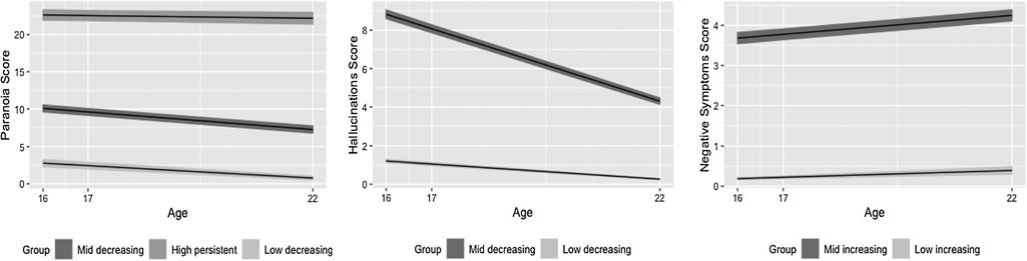
Trajectory plots of best-fitting models for paranoia, hallucinations, and negative symptoms. *Note.* Lines represent mean trajectories; bands represent 95% confidence intervals. Parameter estimates for the trajectories are reported in Table 2.

Model fitting results for converged models are shown in Table 1. Estimated parameters and trajectory plots from the best fitting models are shown in Table 2 and Fig. 1, respectively.

**Table 1.** Growth mixture model fit results for converged models of paranoia, hallucinations, and negative symptoms

*k*	Model	Par.	Constraints	LL	BIC	AIC	Entropy^1^
Paranoia							
1	Model LCGA	5	No growth factor variances	−81 519.705	163 086.393	163 049.410	–
1	Model 0	8	None	−79 987.442	160 050.058	159 990.885	–
2	Model LCGA	11	No growth factor variances	−76 234.770	152 572.904	152 491.540	0.634
2	Model 0	17	None	−75 775.359	151 710.463	151 584.718	0.596
3	Model LCGA	17	No growth factor variances	−74 487.990	149 135.725	149 009.981	0.669
3^a^	Model 0	24	None	−74 316.664	**148 858.850**	148 681.328	0.656
Hallucinations							
1	Model LCGA	5	No growth factor variances	−65 917.295	131 881.575	131 844.589	–
1	Model 0	8	None	−64 673.683	129 422.544	129 363.367	–
2	Model 1A	13	Within-class residual variances	−53 996.157	**108 114.477**	108 018.314	0.776
2	Model 2A	12	Within-class and between-class residual variances	−60 064.952	120 242.670	120 153.904	0.744
2	Model 2B	11	Within-class residual variances and between-class growth factor variances	−54 440.670	108 984.709	108 903.340	0.779
3	Model 2A	18	Within-class and between-class residual variances	−59 209.341	118 587.830	118 454.682	0.768
Negative symptoms							
1	Model LCGA	5	No growth factor variances	−61 820.215	123 687.658	123 650.430	–
1	Model 0	8	None	−59 459.934	118 995.433	118 935.868	–
2	Model LCGA	11	No growth factor variances	−51 529.472	103 162.846	103 080.945	0.790
2	Model 0	17	None	−50 727.482	**101 615.540**	101 488.965	0.788
3	Model 2A	18	Within-class and between-class residual variances	−53 174.429	106 518.879	106 384.859	0.708
3	Model 2C	16	Between-class residual variances and between-class growth factor variances	−54 517.112	109 185.352	109 066.223	0.684

*Note. k*, number of classes; Par., number of estimated parameters for final model; LL, log-likelihood value; AIC, Akaike's information criterion; BIC, Bayesian information criterion. ^1^ = No calculation for 1-class model. Bold typeset indicates lowest BIC value for each dimension. ^a^ = slope factor variance (and covariance) fixed to zero for class #3. Only converged models that were run are shown in this table. All models that were run are shown in online Supplementary Tables S10, S21, S31.

**Table 2.** Parameter estimates of best-fitting model for paranoia, hallucinations, and negative symptoms

*k*	Model	Parameter	Class 1 Mean (s.e.)	*p*	Variance (s.e.)	*p*	Class 2 Mean (s.e.)	*p*	Variance (s.e.)	*p*	Class 3 Mean (s.e.)	*p*	Variance (s.e.)	*p*
Paranoia														
3	Model 0	Intercept	10.075 (0.284)	<0.001	15.564 (1.530)	<0.001	22.639 (0.423)	<0.001	87.410 (7.500)	<0.001	2.786 (0.295)	<0.001	0.119 (0.079)	0.130
		Linear slope	−0.474 (0.025)	<0.001	0.331 (0.061)	<0.001	−0.073 (0.064)	0.257	1.877 (0.214)	<0.001	−0.335 (0.027)	<0.001	0^a^	–
		W1	–	–	19.526 (1.539)	<0.001	–	–	91.913 (7.573)	<0.001	–	–	5.797 (0.723)	<0.001
		W2	–	–	25.816 (2.168)	<0.001	–	–	163.038 (10.676)	<0.001	–	–	4.320 (0.616)	<0.001
		W3	–	–	23.833 (2.729)	<0.001	–	–	190.028 (7.797)	<0.001	–	–	0.406 (0.141)	0.004
		Covariance	–	–	−2.261 (0.259)	<0.001	–	–	−12.193 (1.181)	<0.001	–	–	0^a^	–
Hallucinations														
2	Model 1A	Intercept	8.828 (0.139)	<0.001	25.651 (1.603)	<0.001	1.209 (0.055)	<0.001	1.736 (0.135)	<0.001	–	–	–	–
		Linear slope	−0.754 (0.20)	<0.001	0.237 (0.072)	<0.001	−0.158 (0.007)	<0.001	0.034 (0.002)	<0.001	–	–	–	–
		W1	–	–	27.066 (1.332)	<0.001	–	–	0.189 (0.015)	<0.001	–	–	–	–
		W2	–	–	27.066 (1.332)	<0.001	–	–	0.189 (0.015)	<0.001	–	–	–	–
		W3	–	–	27.066 (1.332)	<0.001	–	–	0.189 (0.015)	<0.001	–	–	–	–
		Covariance	–	–	−2.385 (0.261)	<0.001	–	–	−0.244 (0.017)	<0.001	–	–	–	–
Negative symptoms														
2	Model 0	Intercept	3.682 (0.085)	<0.001	7.089 (0.465)	<0.001	0.189 (0.014)	<0.001	0.031 (0.011)	0.004	–	–	–	–
		Linear slope	0.095 (0.011)	<0.001	0.093 (0.041)	0.022	0.034 (0.008)	<0.001	0.001 (0.001)	0.577	–	–	–	–
		W1	–	–	5.595 (0.418)	<0.001	–	–	0.130 (0.010)	<0.001	–	–	–	–
		W2	–	–	8.220 (0.785)	<0.001	–	–	0.142 (0.013)	<0.001	–	–	–	–
		W3	–	–	8.692 (1.387)	<0.001	–	–	0.356 (0.058)	<0.001	–	–	–	–
		Covariance	–	–	−0.269 (0.069)	<0.001	–	–	0.000 (0.002)	0.819	–	–	–	–

*Note. k*, number of classes; W1–W3, data collection waves 1–3.

Variance of W1–W3 represents residual variance at data collection waves 1–3. Covariance represents covariance between the intercept and slope factors. Model 0: Unconstrained model. Model 1A: Model with within-class residual variances constrained.

a Parameter fixed to zero.

#### Paranoia (online Supplementary Tables S10, S12, S13)

The 3-class Model 0 had the lowest BIC of all best-fitting *k*-class models. Sensitivity analyses suggested better fit of this model compared to the 2-class Models 1A-2C (online Supplementary Table S11), and 3-class homoscedastic model (*df* = 16, loglikelihood = −77 087.757, BIC = 154 325.861, entropy = 0.686). Entropy was notably less than one across all *k*-class models. Most individuals' latent trajectories were characterised by mid-level (56.40%, ‘mid-decreasing’) or low-level (20.66%, ‘low-decreasing’) paranoia scores that decreased over time. For a smaller percentage (22.92%, ‘high-persistent’), paranoia was higher at baseline and persisted over time. Variability around the growth factors was significant for the mid-decreasing and high-persistent latent classes. For the low-decreasing class, slope factor variance was fixed to zero (to avoid singularity of the information matrix), and intercept variability was nonsignificant. Constraining the slope factor means across classes resulted in worse fit (loglikelihood = −74 342.311, BIC = 148 891.350, entropy = 0.651) than the model with freely estimated slopes. Wald tests of the differences between slopes were significant [mid-decreasing, high-persistent: *W* (1) *=* 32.188, *p* < 0.001; mid-decreasing, low-decreasing: *W* (1) *=* 14.139, *p* < 0.001; low-decreasing, high-persistent: *W* (1) *=* 12.704, *p* < 0.001].

#### Hallucinations (online Supplementary Tables S21, S22, S23)

The 2-class Model 1A had the lowest BIC of all best-fitting *k*-class models. Entropy was less than one across all models. The 2-class model suggests a decreasing developmental course of hallucinations across individuals, with significant variability around the growth factors for both classes. Constraining the slope factor means across classes resulted in worse fit (loglikelihood = −54 431.913, BIC = 108 976.593, entropy = 0.763) than the model with freely estimated slopes. For individuals classified in the ‘mid-decreasing’ (45.16%) compared to ‘low-decreasing’ (54.84%) class, hallucinations decreased at a significantly greater rate (*W* (1) = 905.142, *p* < 0.001).

#### Negative symptoms (online Supplementary Tables S31–S33)

The 2-class Model 0 had the lowest BIC of all best-fitting *k*-class models. Entropy was higher for the 2-class models than for the 3-class models. A homoscedastic 2-class model did not fit the data as well (*df* = 12, loglikelihood = −55 901.090, BIC = 111 915.528, entropy = 0.557) as the unconstrained model. The 2-class model suggests an increasing developmental course of negative symptoms across individuals. For a marginal majority (55.07%, ‘mid-increasing’), negative symptoms were estimated at a mid-level at baseline that increased over time. For others (45.93%, ‘low-increasing’), negative symptoms were estimated at a lower initial level that increased over time. There was significant growth factor variability, though slope factor variability was nonsignificant for the low-increasing class. Constraining slope factor means across classes resulted in worse fit (loglikelihood = −50 745.690, BIC = 101 642.510, AIC = 101 523.381, entropy = 0.784) than the model with freely estimated slopes. Negative symptoms increased at a greater rate in the mid-increasing class compared to the low-increasing class [*W* (1) = 18.243, *p* < 0.001].

### Regressions and class-specific means

Whilst we did not identify a ‘persistent’ trajectory for hallucinations, nor for negative symptoms *per se*, we discuss our regression results in terms of the ‘most elevated’ trajectory class (i.e. high-persistent for paranoia, mid-decreasing for hallucinations, and mid-increasing for negative symptoms) for ease of communication.

#### GPS (online Supplementary Tables S15, S25, S35)

In the single-predictor models, the GPSs for clinical help seeking (psychiatrist and general practitioner), major depressive disorder, and attention deficit hyperactivity disorder were significantly associated with increased odds of membership in the most elevated trajectory class compared to the reference class across PENS, as predicted. Similarly, an increase in GPS for years of education (and intelligence, for negative symptoms) was associated with decreased odds for being in the most elevated trajectory class for hallucinations and negative symptoms.

Against predictions, for paranoia, an increase in the GPSs for years of education and intelligence was associated with increased odds for being in the most elevated trajectory class. The GPS for autism spectrum disorder was associated with increased odds of membership in the most elevated trajectory class for paranoia and hallucinations, but not for negative symptoms. The GPSs for schizophrenia, obsessive-compulsive disorder, bipolar disorder, and anorexia were not significantly associated with latent trajectory class membership for any PENS.

In the multiple-predictor models, for paranoia, the GPSs for years of education, intelligence, major depressive disorder, autism spectrum disorder, and attention deficit hyperactivity disorder remained significant, predicting increased odds of membership in the high-persistent class compared to the low-decreasing class. For hallucinations, only the GPS for autism spectrum disorder remained significant. For negative symptoms, only the GPS for years of education remained significant.

#### Family background characteristics (online Supplementary Tables S16, S26, S36)

Family history of schizophrenia and bipolar disorder were both associated with increased odds of being in the most elevated trajectory class for paranoia and hallucinations, but not negative symptoms. As predicted, male sex was associated with membership in the most elevated trajectory class for negative symptoms. As predicted for hallucinations and negative symptoms, lower SES was associated with membership in the most elevated trajectory class. For paranoia, opposite to our hypotheses, higher SES was associated with increased odds of being in the high-persistent trajectory class compared to the low-decreasing class. In the multiple-predictor regressions, all associations remained significant for hallucinations and negative symptoms, and all except the family history of bipolar disorder remained significant for paranoia.

#### Age 7 characteristics (online Supplementary Tables S17, S27, S37)

For all PENS, more life events, and higher emotional and behavioural problems (SDQ scores) were associated with increased odds of being in the most elevated trajectory class, as was lower educational attainment for hallucinations and negative symptoms, as predicted. However, for paranoia, *higher* educational attainment was associated with increased odds of being in the most elevated trajectory class. In the multiple-predictor models, SDQ remained significantly associated with PENS class membership. For paranoia, higher educational attainment also remained significant.

#### Age 22 characteristics

For hallucinations and negative symptoms, class-specific means for educational attainment were lower, and class-specific means for life events, depressive symptoms (MFQ scores) and SDQ scores were higher in the most elevated trajectory class compared to the reference class, as predicted. For paranoia, the same pattern of results was found except that the class-specific mean for educational attainment was *higher* in the high-persistent class compared to the low-decreasing class.

Table 3 also shows the class-specific means for the GPS, background, and age 7 variables. These results mirror the main findings from the multinominal regression analyses.

**Table 3.** Characteristics of latent trajectory classes for paranoia, hallucinations, and negative symptoms

		Paranoia					Hallucinations				Negative symptoms			
Variable	Scaling	*N*	Mean (s.e.)			Diff.	*N*	Mean (s.e.)		Diff.	*N*	Mean (s.e.)		
			Low-dec class (a)	Mid-dec class (b)	High-pers class (c)			Low-dec class (a)	Mid-dec class (b)			Low-inc class (a)	Mid-inc class (b)	Diff.
GPS:														
GPS_EDU_	Std	7090	−0.186 (0.037)	0.058 (0.021)	0.037 (0.028)	a < b, a < c, b = c	7093	0.046 (0.019)	−0.027 (0.020)	a > b	7439	0.157 (0.021)	−0.098 (0.018)	a > b
GPS_IQ_	Std	7090	−0.175 (0.037)	0.030 (0.021)	0.087 (0.030)	a < b, a < c, b = c	7093	−0.012 (0.019)	0.013 (0.020)	a = b	7439	0.059 (0.021)	−0.038 (0.018)	a > b
GPS_PSYCH_	Std	7090	−0.052 (0.038)	−0.041 (0.020)	0.109 (0.029)	a = b, a < c, b < c	7093	−0.044 (0.019)	0.046 (0.020)	a < b	7439	−0.046 (0.021)	0.022 (0.018)	a < b
GPS_GP_	Std	7090	−0.050 (0.039)	−0.060 (0.021)	0.134 (0.027)	a = b, a < c, b < c	7093	−0.059 (0.019)	0.057 (0.019)	a < b	7439	−0.064 (0.021)	0.030 (0.018)	a < b
GPS_SCZ_	Std	7090	0.045 (0.038)	−0.024 (0.021)	0.011 (0.029)	a = b, a = c, b = c	7093	0.004 (0.019)	−0.015 (0.020)	a = b	7439	−0.008 (0.021)	0.013 (0.018)	a = b
GPS_OCD_	Std	7090	−0.041 (0.037)	0.021 (0.021)	−0.031 (0.029)	a = b, a = c, b = c	7093	−0.018 (0.019)	0.011 (0.020)	a = b	7439	−0.021 (0.021)	0.012 (0.018)	a = b
GPS_MDD_	Std	7090	−0.079 (0.037)	0.008 (0.021)	0.034 (0.029)	a = b, a < c, b = c	7093	−0.032 (0.019)	0.031 (0.020)	a < b	7439	−0.056 (0.021)	0.042 (0.018)	a < b
GPS_BIP_	Std	7090	0.008 (0.037)	0.004 (0.021)	−0.014 (0.028)	a = b, a = c, b = c	7093	0.021 (0.019)	−0.019 (0.020)	a = b	7439	0.034 (0.021)	−0.024 (0.018)	a = b
GPS_ASD_	Std	7090	−0.154 (0.036)	0.006 (0.020)	0.098 (0.029)	a < b, a < c, b < c	7093	−0.043 (0.019)	0.055 (0.020)	a < b	7439	−0.026 (0.021)	0.021 (0.018)	a = b
GPS_ANOREX_	Std	7090	−0.034 (0.038)	−0.018 (0.020)	0.049 (0.029)	a = b, a = c, b = c	7093	0.004 (0.019)	−0.006 (0.020)	a = b	7439	0.012 (0.021)	−0.007 (0.018)	a = b
GPS_ADHD_	Std	7090	−0.098 (0.037)	−0.015 (0.021)	0.069 (0.029)	a = b, a < c, b = c	7093	−0.047 (0.019)	0.038 (0.020)	a < b	7439	−0.040 (0.021)	0.027 (0.018)	a < b
Background:														
Sex (male)^1^	1 = male	12 049	0.479 (0.014)	0.439 (0.008)	0.386 (0.011)	a > b, a > c, b > c	12 054	0.451 (0.007)	0.410 (0.008)	a > b	12 652	0.420 (0.008)	0.484 (0.007)	a < b
SES	Std	11 368	−0.030 (0.030)	0.289 (0.018)	0.191 (0.025)	a < b, a < c, b > c	11 373	0.249 (0.017)	0.157 (0.018)	a > b	11 961	0.351 (0.019)	0.124 (0.016)	a > b
Family SCZ^1^	1 = yes	9673	0.021 (0.005)	0.038 (0.004)	0.055 (0.007)	a < b, a < c, b < c	9678	0.035 (0.004)	0.045 (0.004)	a < b	9737	0.035 (0.004)	0.044 (0.004)	a = b
Family BIP^1^	1 = yes	9459	0.045 (0.007)	0.055 (0.005)	0.071 (0.007)	a = b, a < c, b = c	9463	0.050 (0.004)	0.065 (0.005)	a < b	9523	0.052 (0.005)	0.062 (0.005)	a = b
Age 7:														
Ed attainment	Std	7662	−0.108 (0.039)	0.193 (0.020)	0.167 (0.028)	a < b, a < c, b = c	7665	0.185 (0.019)	0.080 (0.019)	a > b	8172	0.243 (0.020)	0.038 (0.018)	a > b
Life events	0–11	9605	0.967 (0.043)	0.946 (0.023)	1.095 (0.035)	a = b, a < c, b < c	9611	0.948 (0.022)	1.037 (0.024)	a < b	10 235	0.944 (0.025)	1.029 (0.022)	a < b
SDQ	0–40	9601	7.445 (0.154)	7.720 (0.088)	8.998 (0.132)	a = b, a < c, b < c	9607	7.553 (0.081)	8.538 (0.089)	a < b	10 231	6.611 (0.082)	9.115 (0.083)	a < b
Age 22:														
Ed attainment	Std	8342	−0.138 (0.040)	0.097 (0.018)	−0.008 (0.026)	a < b, a < c, b > c	8342	0.096 (0.017)	−0.041 (0.019)	a > b	8024	0.239 (0.017)	−0.094 (0.018)	a > b
Life events	0–44	8373	2.170 (0.104)	2.694 (0.062)	5.117 (0.128)	a < b, a < c, b < c	8372	2.606 (0.053)	4.14 (0.079)	a < b	7579	2.705 (0.064)	3.562 (0.070)	a < b
MFQ	0–16	8562	2.215 (0.112)	3.599 (0.070)	7.193 (0.114)	a < b, a < c, b < c	8562	3.382 (0.062)	5.557 (0.075)	a < b	8239	3.495 (0.073)	4.963 (0.069)	a < b
SDQ	0–40	8565	7.075 (0.178)	9.361 (0.099)	14.756 (0.154)	a < b, a < c, b < c	8565	8.915 (0.091)	12.405 (0.105)	a < b	8243	8.903 (0.106)	11.634 (0.098)	a < b

*Note. N* indicates the number of individuals with data contributing to the GMM and not missing on the auxiliary variable. Related and unrelated individuals included, using cluster-robust s.e. For binary variables (^1^), the mean represents the proportion. Diff. reflects the chi-square value (*df* 1) of the difference between the means (or proportions), significant at FDR-adjusted *q* < 0.05 unless indicated by ‘ = ’.

GPS, genome-wide polygenic score; GPS_EDU_, years of education; GPS_IQ_, intelligence; GPS_PSYCH_, ever visited a psychiatrist for nerves, anxiety, tension, or depression; GPS_GP_, ever visited a general practitioner for nerves, anxiety, tension, or depression; GPS_OCD_, obsessive-compulsive disorder; GPS_MDD_, major depressive disorder; GPS_ASD_, autism spectrum disorder; GPS_ADHD_, attention deficit hyperactivity disorder; SES, socioeconomic status; Family SCZ, family history of schizophrenia; Family BIP, family history of bipolar disorder; Ed attainment, educational attainment; SDQ, Strengths and Difficulties Questionnaire total; MFQ, Short Mood and Feeling Questionnaire; Std, standardised.

## Discussion

This study investigated trajectories of paranoia, hallucinations, and negative symptoms from mid-adolescence to emerging adulthood in a community sample. We found evidence to suggest that the developmental distribution of these PENS dimensions was best described by multiple latent classes. Across PENS, trajectory classes identified through GMM were largely distinguished by different scores at age 16, but also by different rates of change over time. We found support for the hypothesis that persistence is associated with less favourable scores on both polygenic and phenotypic behavioural and educational attainment measures.

Of the models selected as providing the best representation of the data, a high and persisting latent trajectory class was identified only for paranoia. The percentage of individuals most likely to be assigned to this class for paranoia (~23%) mirrors the ~20% persistence rate estimated through meta-analysis of aggregated psychotic experiences reported across the lifespan, from studies that manually classified individuals (Linscott & van Os, 2013). Notably, the rate of persistence found in the current study is higher than in previous studies that have estimated trajectories of aggregated psychotic experiences through latent variable modelling, both in adolescence (1–16% for persistent/increasing scores) (Bourque et al., 2017; Lin et al., 2011; Mackie et al., 2011; Thapar et al., 2012; Wigman et al., 2011c), and adulthood (12%) (Wigman et al., 2011a).

Speculatively, these latent variable modelling estimates of persistence may be attenuated in comparison to our paranoia estimate because they include information on hallucinations as well as paranoia/delusions. That is, whilst it was hypothesised that a persistent class would emerge for hallucinations as well as paranoia, in our study, the 2-class model (that did not include a high-persistent class) was selected; suggesting that the data is best represented by a decreasing developmental course across each trajectory class. Considering the empirically driven constraints on the within-class residual variances in this model, and because our study is the first to estimate latent trajectories of hallucinations as a separate dimension in the community, future research should test whether a high-persistent class for paranoia but not hallucinations is replicated in other community samples of young people using other measures.

We also hypothesised that a persistent trajectory would be identified for negative symptoms. In our study, the 2-class model was selected (because of the better relative model fit and entropy), which suggested an overall pattern of increase from adolescence to emerging adulthood. Our results are the first to estimate latent growth in negative symptoms in the community, though they may be considered in-line with findings from a sample of individuals meeting the criteria for a first episode of psychosis, in which most individuals were classified into subgroups characterised by increasing or stable symptoms (Austin et al., 2015). The observed association between male sex and the most elevated negative symptoms trajectory class adds to findings that have reported cross-sectional associations between negative symptoms and male sex (Dominguez et al., 2010; Maric et al., 2003; Ronald et al., 2014).

The findings of higher GPS for clinical help seeking, major depressive disorder, and attention deficit hyperactivity disorder being associated with membership in the most elevated trajectory class across PENS, suggest that the development of PENS dimensions is at least in part associated with measured genetic variants associated with broad clinical outcomes. The association between polygenic liability for clinical help-seeking (for nerves, anxiety, tension, or depression) and for major depressive disorder, can be considered in-line with both theory and empirical findings suggesting that affective symptoms exacerbate psychotic symptoms in general, and specifically, contribute to the persistence of paranoia (Bird, Waite, Rowsell, Fergusson, & Freeman, 2017; Fowler et al., 2012; Freeman & Garety, 2003; Freeman, Garety, Kuipers, Fowler, & Bebbington, 2002; 2012).

Our findings of a null association for schizophrenia GPS concur with those from a recent study of aggregated psychotic experiences measured across adolescence and emerging adulthood (Rammos et al., 2021). To the extent that schizophrenia GPS is associated with PENS measured at single time-points (Jones et al., 2016; Pain et al., 2018), the current results suggest that polygenic liability for schizophrenia may influence the static expression but not the development of PENS. A study that investigated the effect of polygenic liability to schizophrenia GPS on the developmental course of separate dimensions within the negative symptoms construct in schizophrenia found that schizophrenia GPS predicted a more severe course specifically of avolition (Jonas et al., 2019). This aligns broadly with suggestive cross-sectional GPS findings in the community (Havers, Cardno, Freeman, & Ronald, 2022), and with clinical findings and theory suggesting that avolition may be a central feature of the negative symptoms construct (Foussias & Remington, 2010; Strauss et al., 2020, 2021).

Our findings further suggest that family history of psychosis and bipolar disorder, which were associated with the most elevated course of paranoia and hallucinations (though not negative symptoms), is not due to an increased burden of measured polygenic variants for schizophrenia, echoing the findings of Rammos et al. (2021). A range of evidence suggests that psychotic experiences likely reflect a broad transdiagnostic risk, rather than genetic risk for clinical psychosis, specifically (McGorry & Mei, 2021; McGrath et al., 2016).

An unexpected result was that *higher* years of education and *higher* intelligence GPS, as well as higher SES and phenotypic educational attainment at ages 7 and 22, were associated with membership in the high-persistent class for paranoia. These findings are hard to interpret in a theoretical context. Whilst broadly similar associations have been found between these measures and paranoia reported at a single time-point in adulthood (Freeman et al., 2011), our findings may further reflect dimension-specific polygenic and behavioural associations specifically for the development of paranoia, which may otherwise be obscured when aggregated measures are used. Replication in other samples using dimensional PENS scales is required to explore this suggestion further and could further test the extent to which the enforced model constraints, and the observed classification error (reflected in the entropy), may have influenced the unexpected results.

Several strengths and limitations of our study should be highlighted. Key strengths are the estimation of trajectories for separate PENS dimensions using data from a community sample, inherently free of treatment confounds, across a period that reflects when psychotic disorders are likely to first emerge (Kessler et al., 2007; Maibing et al., 2015). Further, utilising full information maximum likelihood allowed for the estimation of trajectories using data from all individuals. Nonetheless, only families already responding at 16 were invited to participate at age 17, and the sample at this age was smaller than at ages 16 and 22. A greater number of repeated measures collected over a greater time-period would allow for a more expansive investigation into nonlinear aspects of growth and provide a broader view of the development of PENS. Further, it is highlighted that whilst the multiple latent classes could be representative of underlying subgroups of individuals, they should primarily be considered as statistical approximations. A final limitation is that with newer, larger GWASs, the polygenic score results may change, and this is an important area for future research.

In summary, we modelled latent heterogeneity in the development of paranoia, hallucinations, and negative symptoms in the community. Studying specific PENS dimensions allowed for distinct patterns of growth to be estimated. Our results suggest largely dimension-wide but also dimension-specific polygenic and behavioural associations with the developmental trajectories of PENS. These findings add to a growing body of literature that suggests that a dimension-specific approach may be important for delineating aetiological and developmental pathways for PENS, which in turn may reduce poor outcomes by facilitating more precise intervention and prevention efforts.

## Supplementary material

For supplementary material accompanying this paper visit https://doi.org/10.1017/S0033291722002914. click here to view supplementary material
