# Supplementary material for: Psychotic experiences and negative symptoms from adolescence to emerging adulthood: developmental trajectories and associations with polygenic scores and childhood characteristics
Source: Psychol Med. 2022 Oct 3;53(12):5685–97. doi: 10.1017/S0033291722002914 (PMC10482726; doi:10.1017/S0033291722002914)
Supplement: Supplementary file 1 [file S0033291722002914sup001.docx]

**Index**

*Information*

**Supplementary Information 1.** Specific Psychotic Experiences Questionnaire (SPEQ) subscales

**Supplementary Information 2.** Additional measures

**Supplementary Information 3.** Genotyping of TEDS participants

**Supplementary Information 4.** Calculation of genome-wide polygenic scores

**Supplementary Information 5.** Growth mixture model post-hoc sensitivity tests

*Figures*

**Supplementary Figure 1.** Linear growth mixture model with individually varying time-scores

**Supplementary Figure 2.** Decision-making flowchart for growth mixture models

**Supplementary Figure 3.** Spaghetti plot of individual trajectories for observed paranoia scores

**Supplementary Figure 4.** Spaghetti plot of individual trajectories for observed hallucinations scores

**Supplementary Figure 5.** Spaghetti plot of individual trajectories for observed negative symptoms scores

*Descriptive statistics*

**Supplementary Table 1.** The Twins Early Development Study (TEDS) sample

**Supplementary Table 2.** Descriptive statistics for paranoia, hallucinations, negative symptoms, and age

**Supplementary Table 3.** Correlations for paranoia, hallucinations, and negative symptoms

**Supplementary Table 4.** Paranoia data time-point characteristics

**Supplementary Table 5.** Hallucinations data time-point characteristics

**Supplementary Table 6.** Negative symptoms data time-point characteristics

*Paranoia*

**Supplementary Table 7.** Longitudinal measurement invariance analysis results for paranoia

**Supplementary Table 8.** Latent growth curve model fit results for results for paranoia

**Supplementary Table 9.** Parameter estimates from linear growth curve model of paranoia

**Supplementary Table 10.** Growth mixture model fit results for paranoia

**Supplementary Table 11**. Sensitivity analysis: Growth mixture model fit results for 2-class constrained models of paranoia

**Supplementary Table 12.** Parameter estimates for each best fitting *k-*class model for paranoia

**Supplementary Table 13.** Most likely class classification values for each best fitting *k-*class model for paranoia

**Supplementary Table 14**. Multinomial logistic regression results for paranoia latent trajectory class regressed on GPSs for all GPS *f*

**Supplementary Table 15.** Multinomial logistic regression results for paranoia latent trajectory class regressed on GPSs

**Supplementary Table 16**. Multinomial logistic regression results for paranoia latent trajectory class regressed on family background variables

**Supplementary Table 17**. Multinomial logistic regression results for paranoia latent trajectory class regressed on age 7 variables

*Hallucinations*

**Supplementary Table 18.** Longitudinal measurement invariance analysis results for hallucinations

**Supplementary Table 19.** Latent growth curve model fit results for results for hallucinations

**Supplementary Table 20.** Parameter estimates from linear growth curve model of hallucinations

**Supplementary Table 21.** Growth mixture model fit results for hallucinations

**Supplementary Table 22.** Parameter estimates for each best fitting *k-*class model for hallucinations

**Supplementary Table 23.** Most likely class classification values for each best fitting *k-*class model for hallucinations

**Supplementary Table 24**. Multinomial logistic regression results for hallucinations latent trajectory class regressed on GPSs for all GPS *f*

**Supplementary Table 25**. Multinomial logistic regression results for hallucinations latent trajectory class regressed on GPSs

**Supplementary Table 26**. Multinomial logistic regression results for hallucinations latent trajectory class regressed on family background variables

**Supplementary Table 27**. Multinomial logistic regression results for hallucinations latent trajectory class regressed on age 7 variables

*Negative symptoms*

**Supplementary Table 28.** Longitudinal measurement invariance analysis results for negative symptoms

**Supplementary Table 29.** Latent growth curve model fit results for results for negative symptoms

**Supplementary Table 30.** Parameter estimates from linear growth curve model of negative symptoms

**Supplementary Table 31.** Growth mixture model fit results for negative symptoms

**Supplementary Table 32.** Parameter estimates for each best fitting *k-*class model for negative symptoms

**Supplementary Table 33.** Most likely class classification values for each best fitting *k-*class model for negative symptoms

**Supplementary Table 34**. Multinomial logistic regression results for negative symptoms latent trajectory class regressed on GPSs for all GPS *f*

**Supplementary Table 35**. Multinomial logistic regression results for negative symptoms latent trajectory class regressed on GPSs

**Supplementary Table 36**. Multinomial logistic regression results for negative symptoms latent trajectory class regressed on family background variables

**Supplementary Table 37**. Multinomial logistic regression results for negative symptoms latent trajectory class regressed on age 7 variables

*References*

**Supplementary Information 1.** Specific Psychotic Experiences Questionnaire (SPEQ) subscales

Subscales and their development are described in full in the Specific Psychotic Experiences Questionnaire paper (Ronald et al., 2014). The subscales were based on existing measures (detailed below) and were adapted for use in adolescents by clinical experts. In the development of the SPEQ, the subscales showed good test-retest reliability (*r* = .65-.68) and internal consistency (α = .85-.93).

*Paranoia* was measured by 15 items adapted from the Paranoia Checklist (Freeman et al., 2005). Individuals were asked how often they have thought, for example, “I can detect coded messages about me in the press/TV/internet”, and “People might be conspiring against me”. Ratings were on a 6-point scale (‘not at all’, ‘rarely’, ‘once a month’, ‘once a week’, ‘several times a week’, ‘daily’).

*Hallucinations* were measured by nine items adapted from the Cardiff Anomalous Perceptions Scale (Bell et al., 2006). Individuals were asked to rate the frequency that they, for example, “Hear sounds or music that people near you don’t hear?”, and “See shapes, lights, or colours even though there is nothing really there?”. Ratings were on a 6-point scale (‘not at all’, ‘rarely’, ‘once a month’, ‘once a week’, ‘several times a week’, ‘daily’).

*Negative symptoms* were measured by eight items adapted from the Scale for the Assessment of Negative Symptoms (Andreasen, 1982). Parents were asked to rate how strongly they agree or disagree with statements such as, “My child often fails to smile or laugh at things others would find funny”, and “My child seems emotionally ‘flat’, for example, rarely changes the emotions he/she shows”. Ratings were on a 4-point scale (‘not at all’, ‘somewhat true’, ‘mainly true’, ‘definitely true’). The following items were not included in the current analyses, in line with current conceptualisations of the negative symptoms construct (see Havers et al., 2022): “My child does not pay attention when being spoken to”, and “My child is often inattentive and appears distracted”.

**Supplementary Information 2.** Additional measures

*Background measures: Socioeconomic status* is a standardised composite of five variables derived from information reported by parents of the TEDS participants at first contact, including mother’s age at birth of first child, mother’s and father’s qualifications and employment. *Family history of schizophrenia / bipolar disorder* was reported by parents at age 16, indicating whether a parent or sibling has schizophrenia or bipolar disorder.

*Age 7 measures: Educational attainment* is a standardised composite of teacher-reported National Curriculum (UK) levels for English and Maths. *Life events* is a total score of 11 specific life events reported by parents over the last three years.

*Age 22 measures: Educational attainment* is a standardised composite derived from information reported by individuals regarding their current studies and qualifications, reflecting probable highest level of qualification after current study and degree classification for those who have already graduated. *Life events* is a total score of eleven specific life events since age 16 (Coddington, 1972).

Items used in these measures are listed in the TEDS study questionnaire booklets, which can be downloaded from <https://www.teds.ac.uk/datadictionary/home.htm>, where calculation of the scores is also described in detail.

**Supplementary Information 3.** Genotyping of TEDS participants

This information is reproduced from Havers et al., 2022 (Supplementary Information 2) under a creative commons licence, with some amendments.

Genotyping of TEDS participants was carried out by other TEDS researchers (Selzam et al., 2018). Full details of the genotyping procedures can be found on the TEDS data dictionary website (https://www.teds.ac.uk/datadictionary/studies/dna.htm).

There have been five phases of genotyping in the TEDS sample since 1998. Data from all phases has contributed towards the ‘genotypic sample’ in TEDS, for which genome-wide polygenic scores were calculated. DNA was collected from cheek swabs between 1998 and 2009 for phases 1-4, and from saliva samples between 2014-2015 for phase 5. Twin pairs (or individual twins) who had recently returned data were prioritised for DNA collection. Families were contacted by mail in phase 1. In phase 2, families were contacted by phone before by mail, following initial verbal consent. In the later phases, families were contacted by mail followed by phone for families who had not responded. Cheek swab samples were collected from individuals by their parents and saliva samples were collected by individuals themselves. Collection was carried out at home and samples were returned by post. The Affymetrix platform was used for the cheek swab samples from phases 1-4 (AffymetrixGeneChip 6.0 SNP arrays). The Illumina Human OEE platform was used for the saliva samples from phase 5 (using OmniExpressExome-8v1.2 arrays). The OEE platform was also used for some cheek swab samples from earlier phases (see <https://www.teds.ac.uk/datadictionary/studies/dna.htm#oee>). Detailed information regarding exclusions can be found on the TEDS data dictionary website (<https://www.teds.ac.uk/datadictionary/studies/dna.htm>); broad exclusions were made on the basis that parents self-reported their ethnic origin as ‘other’ than ‘white’, and where serious medical conditions and or perinatal complications had been self-reported.

The genotypic sample in TEDS includes data from both the Affymetrix and OEE platforms, which were combined and subjected to quality control procedures (described in detail in S1 Methods, Supplementary Methods, Selzam et al., 2018). From an initial combined sample size of 11869, 1523 samples were removed owing to possible non-European ancestry, heterozygosity anomalies, genotype call rate <0.98, and genetic relatedness other than dyzygosity. The final genotypic sample is comprised of 10346 individual twins (3057 genotyped on Affymetrix, 7289 genotyped on OEE). Of the 10346 individuals, there is genotype data from 3320 dyzygotic twin pairs. There are 3706 twin pairs of any zygosity with only one twin genotyped (2666 monozygotic, 1017 dyzygotic and 23 unknown zygosity). There are 7026 twin pairs with either one or both twin genotyped. Seven million (7)363646 genotyped and imputed single nucleotide polymorphisms (SNPs) were retained for subsequent analyses.

**Supplementary Information 4.** Calculation of genome-wide polygenic scores

This information is reproduced from Havers et al., 2022 (Supplementary Information 3) under a creative commons licence, with some amendments.

GPS were calculated by other TEDS researchers (Selzam et al., 2019).

Genome-wide polygenic scores (GPS) were calculated for each of the 10346 individuals in the genotypic sample (see Supplementary Information 3). GPS for years of education (GPS_EDU_) were derived using data from the 2018 GWAS (genome-wide association study) with 23andMe samples removed, comprising N = 766345 (Lee et al., 2018). GPS for intelligence (GPS_IQ_) were derived using data from the 2018 GWAS meta-analysis, comprising N = 266453 (Savage et al., 2018). GPS for visited a psychiatrist for nerves, anxiety, tension, or depression (GPS_PSYCH_), were derived using data from the 2017 GWAS, comprising 64579 cases and 510625 controls (Neale Lab, 2017). GPS for visited a general practitioner for nerves, anxiety, tension, or depression (GPS_GP_), were derived using data from the 2017 GWAS, comprising 192838 cases and 380905 controls (Neale Lab, 2017). GPS for schizophrenia (GPS_SCZ_) were derived using data from the 2018 GWAS, comprising 40675 cases and 64643 controls (Pardiñas et al., 2018). GPS for obsessive compulsive disorder (GPS_OCD_) were derived using data from the 2017 GWAS meta-analysis, comprising 2688 cases and 7037 controls (IOCDF-GC and OCGAS, 2018). GPS for major depressive disorder (GPS_MDD_) were derived using data from the 2018 GWAS meta-analysis (with 23andMe samples removed, comprising 75607 cases and 231747 controls (Wray et al., 2018). GPS for bipolar disorder (GPS_BIP_) were derived using data from the 2011 GWAS, comprising 7481 cases and 9250 controls (Psychiatric GWAS Consortium Bipolar Disorder Working Group, 2011). GPS for autism spectrum disorder (GPS_ASD_) were derived using data from the 2017 GWAS, comprising 18381 cases and 27969 controls (Grove et al., 2019). GPS for anorexia (GPS_ANOREX_) were derived using data from the 2017 GWAS, comprising 3495 cases and 10982 controls (Duncan et al., 2017). GPS for attention deficit hyperactivity disorder (GPS_ADHD_) were derived using data from the 2017 GWAS, comprising 20183 cases and 35191 controls (Demontis et al., 2019). GPS for anxiety (GPS_ANX_) were derived using data from the 2020 GWAS, comprising 26104 cases and 58113 controls (Purves et al., 2020). The description (below) of the methods used for the GPS calculation is adapted directly from Selzam et al. (2019, Supplementary Methods), where the methods are fully described.

GPS are the sum of single nucleotide polymorphisms (SNPs) (individual genetic variants) that are associated with an outcome that are carried by an individual, weighted by the effect sizes of the SNPs. SNP effect sizes are estimated in a genome-wide association study (GWAS) of an outcome of interest in an independent sample (in which the outcome is regressed on each of the SNPs). LDpred software (Vilhjálmsson et al., 2015) was used to calculate the GPS. LDpred implements Bayesian methods, adjusting for linkage disequilibrium (LD) amongst SNPs rather than removing SNPs that are in high LD (as is the case with the clumping and thresholding approach, see, e.g., Choi et al., 2020). LDpred estimates a posterior effect size for each SNP that is present in the GWAS summary statistics as well as in the (target) genotyped sample.

The posterior effect size is estimated as the original summary statistic effect size estimate, adjusted by the relative influence of a SNP (taking into account its level of LD with surrounding SNPs in the target sample) and adjusting for a prior on the effect size of each SNP. A radius corresponding to a 2 megabase window on average around each SNP of interest was set to account for LD. The effect size prior is dependent on the SNP-heritability of the GWAS outcome of interest, and the proportion of SNPs (the fraction of causal markers) believed to influence the outcome. Using the effect size prior, the beta effect sizes are reweighted. Thus, the effects are spread among the SNPs across the genome in proportion to the amount of LD amongst them. The genotype dataset was reduced to SNPs that had imputation quality information scores of 1 to reduce computational demands, resulting in 515100 SNPs that could be analysed. Alleles associated with the outcome were counted for each individual (0, 1, or 2 for each SNP). The GPS for each individual was calculated as the sum of the alleles, each weighted by the posterior SNP effect size.

The first 10 principal components (PCs) were calculated using data from the final genotyped sample, and GPSs were regressed on these PCs prior to analysis. These PCs reflect and capture population structure within the sample. Regressing the GPSs on the principal components adjusts for any confounding that would otherwise be present due to population structure. GPSs were also regressed on batch and chip type to further remove any potential confounding by these variables. Standardized residuals were used in the GPS analyses.

GPS derived from three fractions (*f*) of causal markers (1, 0.3, 0.01) are available to TEDS researchers.

**Supplementary Information 5.** Growth mixture model post-hoc sensitivity tests

Two sets of post-hoc sensitivity tests were conducted. One, to test a more parsimonious parameterisation of the data – where the *k-*1-class model of the overall best fitting *k-*class model was unconstrained (i.e., Model 0), models 1A-2C for *k-*1 were also run. A homoscedastic model (Model 2C) was also run if the best fitting *k­*-class model was unconstrained (and further constrained models had not already been run).

Two, to test for the significance of the difference between the slopes – where the slopes of the latent classes in the overall best fitting model appeared visually parallel, two tests were run: i) an equivalent model with constrained slope factor means, and ii) Wald tests of the differences between the slopes. Better fit of the original model (compared to the model with the constrained slope factor means), and a significant Wald test statistic (*W*, which is chi-square distributed) would imply that the difference between the slope factor means is significant – suggesting that the latent classes differ in terms of the magnitude of their slopes.

**Supplementary Figure 1.** Linear growth mixture model with individually varying time-scores

*Note:* Figurative (simplified) representation of a linear growth mixture model with individually varying time-scores. Boxes represent observed variables; circles represent latent variables; curved arrows represent (co)variances; straight arrows represent regression paths; the triangle represents a constant; diamonds represent definition variables (reflecting individual time-scores/times of measurement). The ‘c’ latent variable represents the categorical latent class variable, which moderates the model parameters within the box. *b_0_* is a continuous latent intercept factor, and *b_1_* is a continuous slope factor. *y* represents the observed score at times 1, 2, and 3, for individual *i.* Residual terms (*e*) are individual-specific and residual variances are wave-specific.

1

1

1

1

*e_i3_*

*e_i2_*

*e_i1_*

*t_i_*_1_

*t_i_*_2_

*t_i_*_3_

1

1

1

*y_i3_*

*y_i2_*

*y_i1_*

*b_1_*

*b_0_*

c

**Supplementary Figure 2.** Decision-making flowchart for growth mixture models, based on information from the Mplus forum (<http://www.statmodel.com/discussion/messages/board-topics>) and from Mplus product support

*Yes*

*Do not report solution*

*No*

Loglikelihood value replicated at least twice, model terminated normally, standard errors calculated, plausible parameter values

*Yes*

*Re-run using the (2x) seed values of the replicated loglikelihood values*

*Report solution*

*Yes*

**Estimated covariance matrix not-positive definite**: Fix parameter/s as per output instructions

**Saddle point reached:** Increase number of expectation step iterations (50000) and reduce convergence criteria (0.000001)

**Insufficient number of expectation step iterations:** Increase number of expectation step iterations (1000)

**Parameters fixed during estimation:** Make adjustments detailed in output of ‘second run’ and request start values, and or proceed to ‘final run’

**Final run**

*k*1-3: Increase to initial stage starts (400), final stage optimizations (100) and initial stage iterations (100). *k*4: No further adjustments

**Second run**

*k*1-3: Increase initial stage starts (100), final stage optimizations (20) and initial stage iterations (100). *k*4: Increase to initial stage starts (400), final stage optimizations (100) and initial stage iterations (100)

*If parameters were fixed, re-run with start values from previous output (with 0 stage starts). Check classes and parameters are replicated*

Loglikelihood value replicated at least twice, model terminated normally, standard errors calculated, plausible parameter values

*No*

Loglikelihood value replicated at least twice, model terminated normally, standard errors calculated, plausible parameter values

**Initial run**

*k*1-3: Initial stage starts 20. *k*4: 100

*k*1-3: Final stage optimizations 4. *k*4: 20

*k*1-3: Initial stage iterations 10. *k*4: 100

*No*

**Supplementary Figure 3.** Spaghetti plot of individual trajectories for observed paranoia scores

**
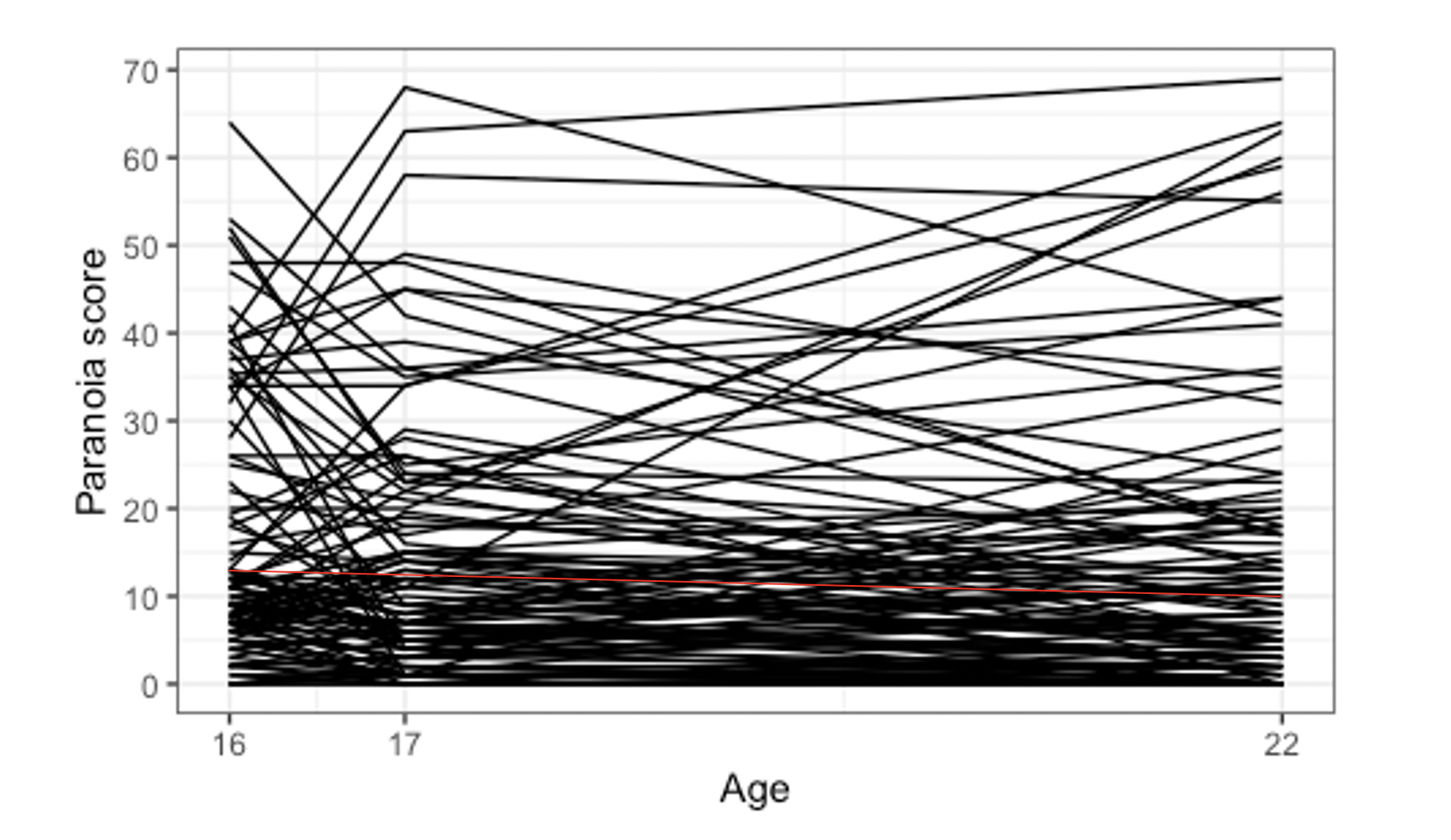
**

*Note:* Individual trajectories are shown for a random draw of 100 individuals with complete data (seed 20). Mean trajectory estimated using a linear growth model across the whole sample (*N* **=** 12,051), plotted in red. Parameter estimates for the mean trajectory reported in Supplementary Table 9.

**Supplementary Figure 4.** Spaghetti plot of individual trajectories for observed hallucinations scores

**
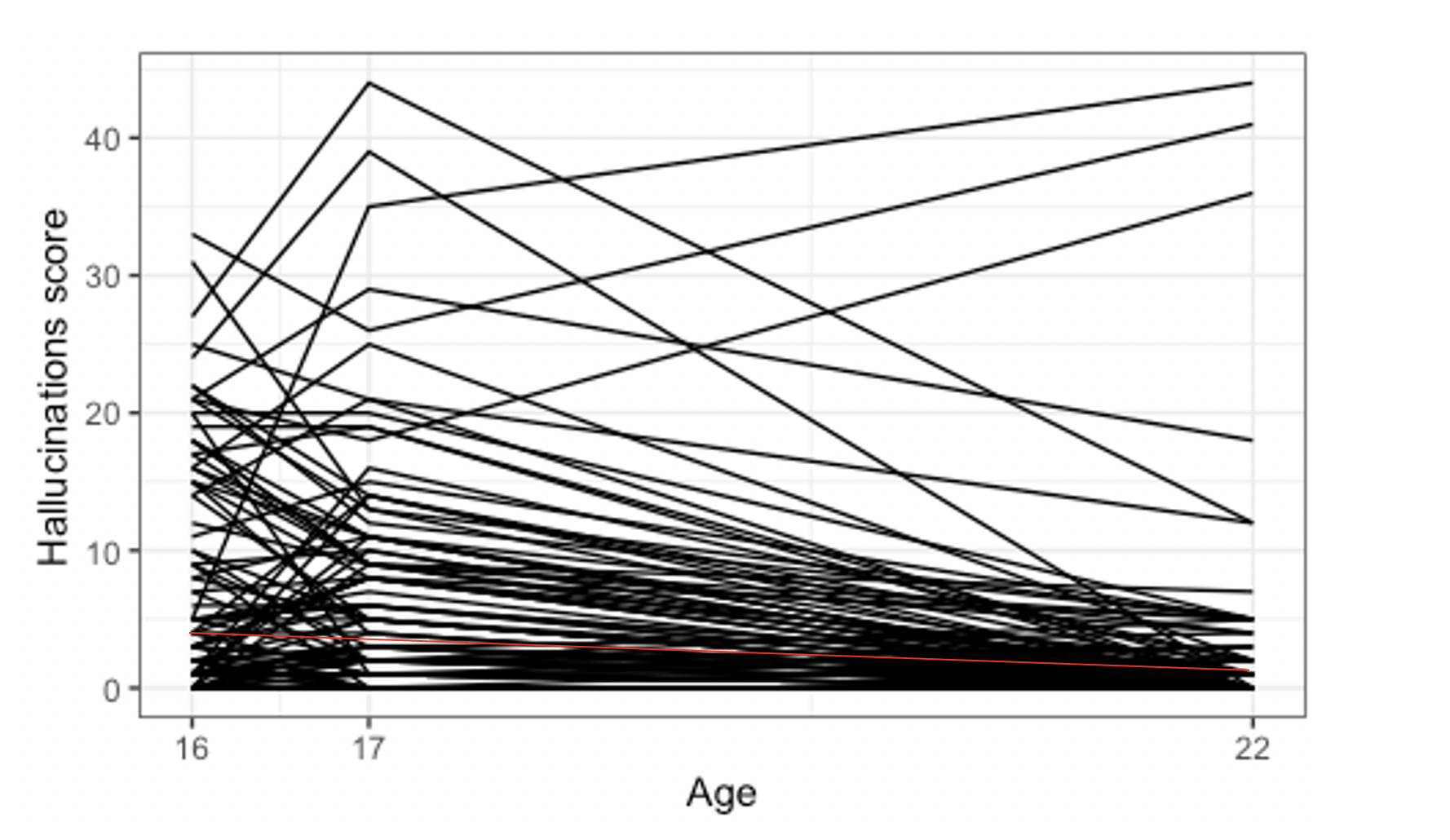
**

*Note.* Individual trajectories are shown for a random draw of 100 unrelated individuals with complete data (seed 20). Mean trajectory estimated using a linear growth model across the whole sample (*N* **=** 12,056), plotted in red. Parameter estimates for the mean trajectory reported in Supplementary Table 20.

**Supplementary Figure 5.** Spaghetti plot of individual trajectories of negative symptoms scores

**
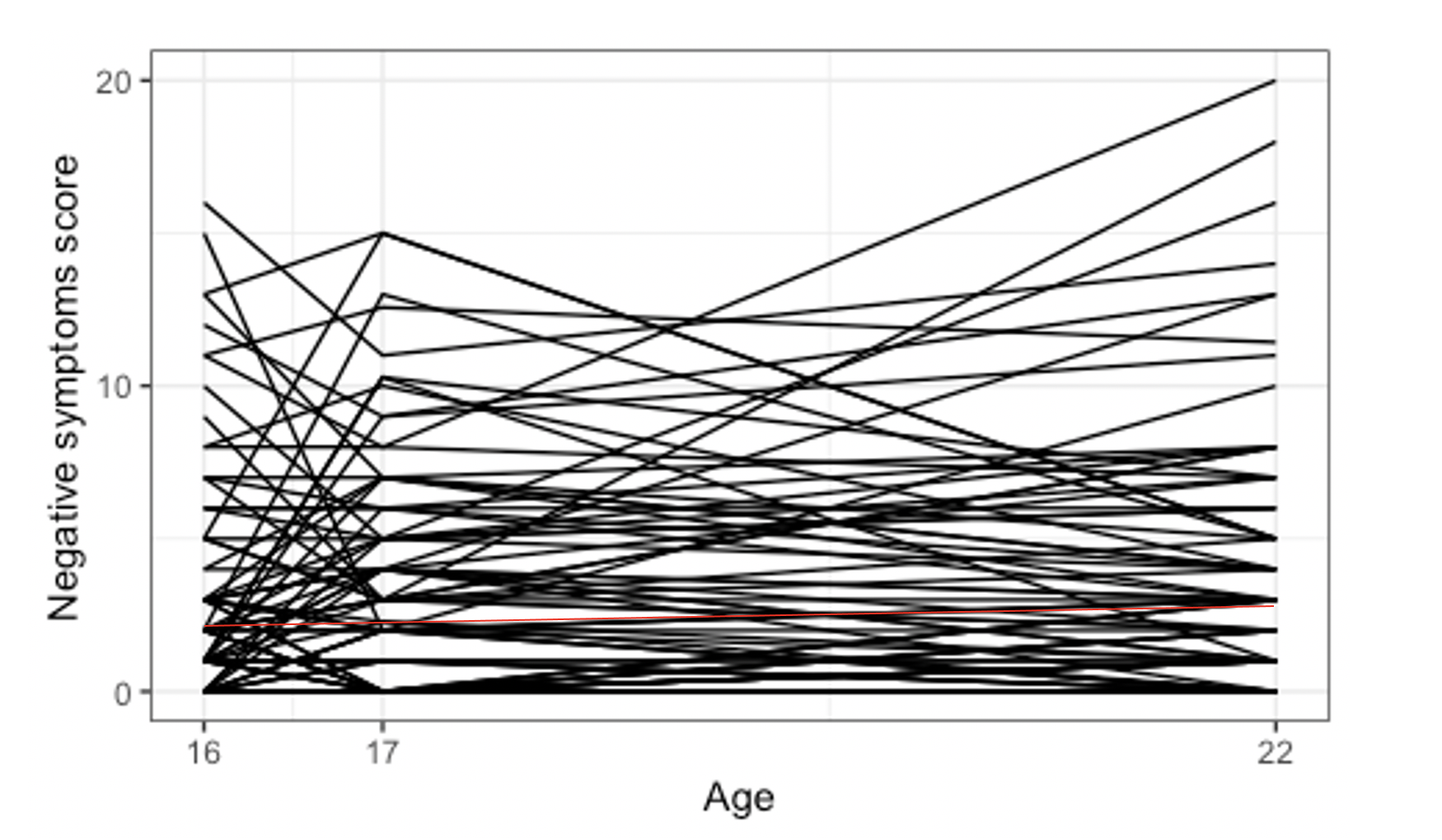
**

*Note.* Individual trajectories are shown for a random draw of 100 individuals with complete data (seed 20). Mean trajectory estimated using a linear growth model across the whole sample (*N* **=** 12,662), plotted in red. Parameter estimates for the mean trajectory reported in Supplementary Table 30.

**Supplementary Table 1.** The Twins Early Development Study (TEDS) sample

This table is reproduced from Havers et al., 2022 (Supplementary) under a creative commons licence, with some amendments.

|  | *N* families contacted | *N* families returned data | % return rate | *N* (approximate) not contacted from ONS sample owing to exclusions ^1^ |
| --- | --- | --- | --- | --- |
| *N* families that responded to initial ONS invitation,  *N =* 16810 (‘ONS sample’) |  |  |  |  |
| 1^st^ contact study | 16302 | 13488 | 82.74% | 500 |
| 16-year study | 10874 | 5123 | 47.11% | 5900 |
| 17-year study | 1773 | 1475 | 83.19% | See ^2^ below |
| 22-year study ^a,b^ | 10451 | 5352 | 51.21% | 6250 |
| 22-year study ^a,c^ | 8611 | 5184 | 60.20% | 8210 |

*Note.* ^a^ ‘22-year study’ is the called the 21-year study on the TEDS website. ^b^ Parent-rated data. ^c^ Twin self-rated data. ^1^ Exclusions were due to families withdrawing from the study, address problems, severe medical conditions, families being inactive, families with no recent data, and for ‘other reasons’, which are detailed in full on the TEDS data dictionary (<https://www.teds.ac.uk/datadictionary/studies/returns/samples.htm>). ^2^ The sample at 17 was a selected subset of 1773 of the families who had returned data at 16.

**Supplementary Table 2.** Descriptive statistics for paranoia, hallucinations, negative symptoms, and age

|  | Paranoia | | |  | Hallucinations | | | Negative symptoms | | |
| --- | --- | --- | --- | --- | --- | --- | --- | --- | --- | --- |
|  | Age 16 | Age 17 | Age 22 | | Age 16 | Age 17 | Age 22 | Age 16 | Age 17 | Age 22 |
| *N* for PENS data | 9,898 | 2,937 | 8,340 | | 9,907 | 2,940 | 8,338 | 9,944 | 2,939 | 10,355 |
| Mean PENS (*SD*) | 12.12 (10.63) | 14.44 (13.64) | 10.09 (11.51) | | 4.72 (6.11) | 6.74 (7.57) | 1.72 (4.13) | 2.19 (3.19) | 2.91 (3.94) | 2.64 (3.60) |
| PENS range | 0-72 | 0-75 | 0-74 | | 0-45 | 0-45 | 0-44 | 0-24 | 0-24 | 0-24 |
| Skewness | 1.60 | 1.46 | 1.87 | | 2.12 | 1.53 | 4.46 | 2.41 | 2.09 | 2.25 |
| *N* >3 *SD* | 121 (1.22%) | 34 (1.16%) | 143 (1.71%) | | 157 (1.58%) | 30 (1.02%) | 153 (1.83%) | 191 (1.92%) | 61 (2.08%) | 206 (1.99%) |
| Coefficient α | 0.93 | 0.95 | 0.94 | | 0.88 | 0.90 | 0.87 | 0.83 | 0.87 | 0.84 |
|  |  | | | |  | | |  | | |
| *N* for age data | 9,922 | 2,963 | 8,508 | | 9,928 | 2,963 | 8,507 | 9,979 | 2,966 | 10,418 |
| Mean age (*SD*) | 16.32 (0.69) | 17.06 (0.88) | 22.85 (0.88) | | 16.32 (0.68) | 17.06 (0.88) | 22.86 (0.88) | 16.32 (0.68) | 17.06 (0.88) | 22.30 (0.93) |
| Age range (years) | 14.91-21.34 | 15.55-19.00 | 21.16-25.19 | | 14.91-21.34 | 15.55-19.00 | 21.16-25.19 | 14.91-19.45 | 15.55-19.00 | 20.56-25.59 |
| Skewness | -0.27 | 0.01 | 0.02 | | -0.27 | 0.01 | 0.02 | -0.30 | -0.01 | 0.13 |

*Note.* PENS = psychotic experiences and negative symptoms**Supplementary Table 3.** Correlations between paranoia, hallucinations, and negative symptoms

|  | Para age 16 | Para age 17 | Para age 22 | Halls age 16 | Halls age 17 | Halls age 22 | NS age 16 | NS age 17 |
| --- | --- | --- | --- | --- | --- | --- | --- | --- |
| Para age 17 | **0.68 [0.65, 0.71]** |  |  |  |  |  |  |  |
| Para age 22 | **0.42 [0.39, 0.45]** | **0.55 [0.50, 0.59]** |  |  |  |  |  |  |
| Halls age 16 | 0.47 [0.45, 0.49] | 0.49 [0.45, 0.53] | 0.26 [0.23, 0.29] |  |  |  |  |  |
| Halls age 17 | 0.40 [0.35 0.44] | 0.55 [0.52, 0.59] | 0.29 [0.23, 0.34] | **0.66 [0.63, 0.68]** |  |  |  |  |
| Halls age 22 | 0.20 [0.16, 0.23] | 0.33 [0.28, 0.39] | 0.36 [0.33, 0.38] | **0.33 [0.26, 0.36]** | **0.42 [0.37, 0.47]** |  |  |  |
| NS age 16 | 0.13 [0.10, 0.15] | 0.16 [0.10, 0.20] | 0.11 [0.07, 0.14] | 0.11 [0.08, 0.14] | 0.13 [0.08, 0.18] | 0.11 [0.08, 0.15] |  |  |
| NS age 17 | 0.19 [0.14, 0.24] | 0.19 [0.13, 0.23] | 0.16 [0.10, 0.22] | 0.15 [0.10, 0.20] | 0.15 [0.10, 0.20] | 0.08 [0.02, 0.14] | **0.69 [0.66, 0.72]** |  |
| NS age 22 | 0.07 [0.04, 0.10] | 0.11 [0.05, 0.16] | 0.12 [0.09, 0.16] | 0.08 [0.05, 0.11] | 0.11 [0.06, 0.17] | 0.12 [0.09, 0.15] | **0.51 [0.48, 0.53]** | **0.57 [0.53, 0.61]** |

*Note. N* = 989-5177 (one randomly selected twin per pair). Para = paranoia. Halls = hallucinations. NS = negative symptoms. Spearman’s rank correlation coefficient [95% confidence intervals]. Bold typeset indicates within-trait correlations.

**Supplementary Table 4.** Paranoia data time-point characteristics

|  | One time-point | | | Two time-points | | Three time-points | Total |
| --- | --- | --- | --- | --- | --- | --- | --- |
|  | Age 16 only | Age 17 only | Age 22 only | Age 16 and 17 only | Age 16 and 22 only | Age 16, 17 and 22 |  |
| *N* | 2745 (22.78%) | 0 (0%) | 2150 (17.85%) | 966 (8.02%) | 4219 (35.02%) | 1968 (16.33%) | 12051 |
| SES (SD) | -0.10 (0.98) | NA | 0.09 (1.01) | 0.07 (0.99) | 0.33 (0.98) | 0.25 (0.98) | NA |
| Female | 41.09% | 0% | 62.98% | 44.82% | 63.43% | 64.02% | NA |
| Genotyped | 1464 (20.65%) | 0 (0%) | 1107 (15.62%) | 541 (7.64%) | 2702 (38.12%) | 1275 (17.99%) | 7089 (58.82%) |

*Note. N* = number of individuals with paranoia total score data across data collection waves. SES = socioeconomic status. NA = not applicable.

**Supplementary Table 5.** Hallucinations data time-point characteristics

|  | One time-point | | | Two time-points | | Three time-points | Total |
| --- | --- | --- | --- | --- | --- | --- | --- |
|  | Age 16 only | Age 17 only | Age 22 only | Age 16 and 17 only | Age 16 and 22 only | Age 16, 17 and 22 |  |
| *N* | 2750 (22.81%) | 1 (0.1%) | 2148 (17.82%) | 967 (8.02%) | 4218 (34.99%) | 1972 (16.36%) | 12056 |
| SES (SD) | -0.10 (0.98) | -1.23 (NA) | 0.09 (1.01) | 0.07 (0.99) | 0.33 (0.98) | 0.25 (0.98) | NA |
| Female | 41.05% | 0% | 63.04% | 44.88% | 63.39% | 64.05% | NA |
| Genotyped | 1467 (20.68%) | 0 (0%) | 1106 (15.59%) | 543 (7.66%) | 2701 (38.08%) | 1276 (17.99%) | 7093 (58.83%) |

*Note. N* = number of individuals with hallucinations total score data across data collection waves. SES = socioeconomic status. NA = not applicable.

**Supplementary Table 6.** Negative symptoms data time-point characteristics

|  | One time-point | | | Two time-points | | Three time-points | Total |
| --- | --- | --- | --- | --- | --- | --- | --- |
|  | Age 16 only | Age 17 only | Age 22 only | Age 16 and 17 only | Age 16 and 22 only | Age 16, 17 and 22 |  |
| *N* | 1762 (13.92%) | 1 (0.1%) | 2717 (21.46%) | 544 (4.30%) | 5244 (41.42%) | 2394 (18.91%) | 12662 |
| SES (SD) | -0.18 (0.95) | -0.06 (NA) | 0.18 (1.02) | -0.22 (0.98) | 0.38 (0.96) | 0.29 (0.96) | NA |
| Female | 47.62% | 0% | 50.42% | 54.60% | 56.50% | 58.60% | NA |
| Genotyped | 880 (11.82%) | 1 (0.1%) | 1437 (19.49%) | 286 (3.62%) | 3310 (44.73%) | 1529 (20.74%) | 7443 (58.78%) |

*Note. N* = number of individuals with negative symptoms total score data across data collection waves. SES = socioeconomic status. NA = not applicable.

**Supplementary Table 7.** Longitudinal measurement invariance analysis results for paranoia

|  | Parameters | Fit indices | | | Comparison of fit indices between nested models | | |
| --- | --- | --- | --- | --- | --- | --- | --- |
|  |  | CFI | RMSEA [90% CI] | SRMR | Δ CFI | Δ RMSEA | Δ SRMR |
| Configural invariance model (no constraints)^a^ | 276 | 0.940 | 0.028 [0.027, 0.029] | 0.035 | - | - | - |
| Metric invariance model (factor loadings constrained)^a^ | 256 | 0.936 | 0.028 [0.028, 0.029] | 0.041 | 0.004 | 0.000 | -0.006 |
| Scalar invariance model (factor loadings and intercepts constrained)^a^ | 237 | 0.922 | 0.031 [0.030, 0.032] | 0.044 | 0.014 | -0.003 | -0.003 |
| Partial scalar invariance model (factor loadings and intercepts constrained) ^a, b^ | 241 | 0.926 | 0.030 [0.029, 0.031] | 0.042 | 0.010 ^c^ | -0.002 ^c^ | -0.001^c^ |
| Partial strict invariance model (factor loadings, intercepts and residual variances constrained) ^a, b^ | 218 | 0.913 | 0.032 [0.032, 0.033] | 0.053 | 0.013 | -0.002 | -0.009 |

*Note. N =* 6,032 (one randomly selected twin per pair). CFI = comparative fit index. RMSEA = root mean square error of approximation. SRMR = standardized root mean square residual. Δ denotes change value. ^a^ Correlated residual variance between item 2 and item 8. ^b^ Item 12 parameters free to vary. ^c^ Change values compared to metric invariance model. The measurement model was a 5-factor model (age 16: χ^2^ (82) = 1,460.301, *P<* .001, CFI = 0.94, RMSEA = 0.08 [0.08, 0.09], SRMR = 0.04; age 17: χ^2^ (82) = 806.819, *P<* .001, CFI = 0.91, RMSEA = 0.11 [0.10, 0.12], SRMR = 0.05; age 22: χ^2^ (82) = 1,248.10, *P<* .001, CFI = 0.94, RMSEA = 0.09 [0.00, 0.09], SRMR = 0.04). Confirmatory factor analysis results available in full from corresponding author.

**Supplementary Table 8.** Latent growth curve model fit results for paranoia

|  | Par. | LL | AIC | BIC | χ^2^ value (*df*) | CFI | RMSEA [90% CI] | SRMR |
| --- | --- | --- | --- | --- | --- | --- | --- | --- |
| Intercept-only model  **Linear growth model** ^a^  Quadratic growth model ^b^  Latent basis growth model ^c^ | 5  **8**  7  7 | -80247.673  **-79983.474**  -80092.438  -79982.140 | 160505.345  **159982.947**  160198.877  159980.279 | 160542.330  **160042.123**  160250.655  160039.454 | 352.740 (4), *p* <.001  **17.657 (1), *p* <.001**  134.876 (2), *p* <.001  10.527 (1), *p* = .001 | 0.823  **0.996**  0.925  0.997 | 0.105 [0.096, 0.115]  **0.033 [0.020, 0.047]**  0.097 [0.084, 0.112]  0.031 [0.021, 0.051] | 0.088  **0.018**  0.085  0.021 |

*Note. N =* 12,051. Related and unrelated individuals included, using cluster-robust SE. LL = loglikelihood value. AIC = Akaike’s Information Criterion. BIC = Bayesian Information Criterion. χ^2^ = chi-square value. CFI = comparative fit index. RMSEA = root mean square error of approximation. SRMR = standardized root mean square residual. ^a^ Residual variances were freely estimated at each time-point. ^b^ Quadratic slope variance-covariance parameters not estimated, and residual variances constrained to equality (to achieve identification and over-identification, respectively). ^c^ A proper solution could not be obtained for an over-identified latent basis model (with residual variances constrained to equality). Removal of the age 22 residual variance was required to obtain a proper solution of the over-identified model. Bold typeset indicates model that was selected for subsequent growth mixture modelling.

**Supplementary Table 9.** Parameter estimates from linear growth curve model of paranoia

|  | Mean | | | | |  | |  | |  | Variance |  | |  |
| --- | --- | --- | --- | --- | --- | --- | --- | --- | --- | --- | --- | --- | --- | --- |
|  | Estimate | SE | | *z* | | *p* | |  | | Estimate | SE | *z* | | *p* |
| Intercept  Slope  16 years  17 years  22 years | 12.24  -0.37  -  -  - | 0.12  0.02  -  -  - | | 101.14  -15.14  -  -  - | | <.001  <.001  -  -  - | |  | | 83.99  3.01  29.50  79.66  8.06 | 3.22  0.57  2.74  4.73  18.63 | 26.07  5.27  10.76  16.85  0.43 | | <.001  <.001  <.001  <.001  0.67 |
|  | Estimate | | SE | | *z* | | *p* | | Standardised  estimate | |  |  |  |  |
| Factor covariance | -5.68 | | 0.54 | | -10.60 | | <.001 | | -0.36 | |  |  |  |  |

*Note: N =* 12,051. Related and unrelated individuals included, using cluster-robust SE. Unstandardised estimates (unless otherwise indicated).

**Supplementary Table 10.** Growth mixture model fit results for paranoia

| *k* | Model | Par. | Constraints | LL | BIC | AIC | Entropy ^1^ |
| --- | --- | --- | --- | --- | --- | --- | --- |
| 1  1  2  2  3  3  4  4  4  4  4  4  4  4 | Model LCGA  Model 0  Model LCGA  Model 0  Model LCGA  Model 0  Model LCGA  Model 0  Model 1A  Model 1B  Model 1C  Model 2A  Model 2B  Model 2C | 5  8  11  17  17  24  23  35  27  27  29  24  21  20 | No growth factor variances  None  No growth factor variances  None  No growth factor variances  None  No growth factor variances  None  Within-class residual variances  Between-class residual variances  Between-class growth factor variances  Within-class and between-class residual variances  Within-class residual variances and between-class growth factor variances  Between-class residual variances and between-class growth factor variances | -81519.705  -79987.442  -76234.770  -75775.359  -74487.990  -74316.664  -  -  -  -  -  -  -  - | 163086.393  **160050.058**  152572.904  **151710.463**  149135.725  **148858.850**  -  -  -  -  -  -  -  - | 163049.410  159990.885  152491.540  151584.718  149009.981  148681.328  -  -  -  -  -  -  -  - | -  -  0.634  0.596  0.669  0.656  -  -  -  -  -  -  -  - |
|  |  |  |  |  |  |  |  |

*Note. k* = number of classes. Par. = number of estimated parameters (for final model if converged, for unadjusted model if not converged). LL = loglikelihood value. AIC = Akaike’s Information Criterion. BIC = Bayesian Information Criterion. ^1^ = No calculation for 1-class model. Bold typeset indicates lowest BIC value for each *k*-class model. Loglikelihood values replicated for best-fitting *k-*class models using the two random seed values with the highest loglikelihoods.

**Supplementary Table 11**. Growth Mixture Model Fit Results for 2-Class Models of Paranoia (Sensitivity Analysis)

| *k* | Model | Par. | Constraints | LL | BIC | AIC | Entropy |
| --- | --- | --- | --- | --- | --- | --- | --- |
| 2  2  2  2  2  2 | 1A  1B  1C  2A  2B  2C | 13  14  15  12  11  12 | Within-class residual variances  Between-class residual variances  Between-class growth factor variances  Within-class and between-class residual variances  Within-class residual variances and between-class growth factor variances  Between-class residual variances and between-class growth factor variances | -75820.928  -76433.696  -75859.851  -76969.025  -75886.302  -77924.647 | 151764.013  152998.946  151860.654  154050.811  151875.967  155962.054 | 151667.855  152895.392  151749.703  153962.050  151794.603  155873.293 | 0.600  0.580  0.617  0.632  0.617  0.496 |

*Note. k* = number of classes. Par. = number of estimated parameters (initial run). LL = loglikelihood value. AIC = Akaike’s Information Criterion. BIC = Bayesian Information Criterion.

**Supplementary Table 12.** Parameter estimates for each best fitting *k-*class model for paranoia

| *k* | Model | Parameter | Class 1  Mean (SE) | *P* | Variance (SE) | *P* | Class 2  Mean (SE) | *P* | Variance (SE) | *P* | Class 3  Mean (SE) | *P* | Variance (SE) | *P* |
| --- | --- | --- | --- | --- | --- | --- | --- | --- | --- | --- | --- | --- | --- | --- |
| 1 | Model 0 | Intercept  Linear slope  W1  W2  W3  Covariance | 12.362 (0.125)  -0.335 (0.022)  -  -  -  - | <.001  <.001  -  -  -  - | 80.615 (3.077)  0.373 (0.275)  36.480 (2.648)  75.924 (4.924)  89.665 (11.341)  -3.998 (0.463) | <.001  .175  <.001  <.001  <.001  <.001 | -  -  -  -  -  - | -  -  -  -  -  - | -  -  -  -  -  - | -  -  -  -  -  - | -  -  -  -  -  - | -  -  -  -  -  - | -  -  -  -  -  - | -  -  -  -  -  - |
| 2 | Model 0 | Intercept  Linear slope  W1  W2  W3  Covariance | 20.458 (0.400)  -0.142 (0.052)  -  -  -  - | <.001  .006  -  -  -  - | 87.372 (6.170)  1.472 (0.173)  78.336 (6.203)  146.201 (9.415)  169.488 (7.580)  -10.362 (0.975) | <.001  <.001  <.001  <.001  <.001  <.001 | 7.654 (0.161)  -0.458 (0.023)  -  -  -  - | <.001  <.001  -  -  -  - | 17.929 (1.167)  0.215 (0.046)  12.397 (0.991)  14.340 (1.153)  13.739 (1.706)  -1.708 (0.162) | <.001  <.001  <.001  <.001  <.001  <.001 | -  -  -  -  -  - | -  -  -  -  -  - | -  -  -  -  -  - | -  -  -  -  -  - |
| 3 | Model 0 | Intercept  Linear slope  W1  W2  W3  Covariance | 10.075 (0.284)  -0.474 (0.025)  -  -  -  - | <.001  <.001  -  -  -  - | 15.564 (1.530)  0.331 (0.061)  19.526 (1.539)  25.816 (2.168)  23.833 (2.729)  -2.261 (0.259) | <.001  <.001  <.001  <.001  <.001  <.001 | 22.639 (0.423)  -0.073 (0.064)  -  -  -  - | <.001  .257  -  -  -  - | 87.410 (7.500)  1.877 (0.214)  91.913 (7.573)  163.038 (10.676)  190.028 (7.797)  -12.193 (1.181) | <.001  <.001  <.001  <.001  <.001  <.001 | 2.786 (0.295)  -0.335 (0.027)  -  -  -  - | <.001  <.001  -  -  -  - | 0.119 (0.079)  0^a^  5.797 (0.723)  4.320 (0.616)  0.406 (0.141)  0^a^ | .130  -  <.001  <.001  .004  - |

*Note. k* = number of classes. W1-W3 = data collection waves 1-3. Variance of W1-W3 represents residual variance at data collection waves 1-3. Covariance represents covariance between intercept and slope. Model 0: Unconstrained model. ^a^ = parameter manually fixed to zero.

**Supplementary Table 13.** Most likely class classification values for each best fitting *k-*class model for paranoia

| *k* | Model |  | Classification probabilities | | | | Final class counts and proportions |
| --- | --- | --- | --- | --- | --- | --- | --- |
|  |  |  | Class 1 | Class 2 | Class 3 |  |  |
| 1 | Model 0 | Class 1 | 1.000 | - | - |  | 12049 (100%) |
| 2 | Model 0 | Class 1  Class 2 | 0.774  0.046 | 0.226  0.954 | -  - |  | 3766 (31.26%)  8283 (68.74%) |
| 3 ^a^ | Model 0 | Class 1  Class 2  Class 3 | 0.881  0.231  0.117 | 0.039  0.749  0.000 | 0.081  0.020  0.883 |  | 6798 (56.40%)  2762 (22.92%)  2489 (20.66%) |

*Note. k* = number of classes. Model 0: Unconstrained model. ^a^ = constrained slope factor variance in class#3. Values based on most likely latent class membership.

**Supplementary Table 14**. Multinomial logistic regression results for paranoia latent trajectory class regressed on GPSs for all GPS *f*

|  | Beta | | Odds Ratio | | |
| --- | --- | --- | --- | --- | --- |
|  | *b* (SE) | Z (P value) | OR (SE) | 95% CI lower bound | 95% CI upper bound |
| EA3_1 |  |  |  |  |  |
| Low-dec vs mid-dec | 0.244 (0.045) | 5.389 (<.001) | 1.277 (0.058) | 1.168 | 1.396 |
| Low-dec vs high-persistent | 0.223 (0.046) | **4.816 (<.001)** | 1.250 (0.058) | 1.142 | 1.369 |
| Mid-dec vs high-persistent | -0.021 (0.038) | -0.550 (.583) | 0.979 (0.037) | 0.909 | 1.055 |
|  |  |  |  |  |  |
| EA3_0.3 |  |  |  |  |  |
| Low-dec vs mid-dec | 0.234 (0.046) | 5.094 (<.001) | 1.264 (0.058) | 1.155 | 1.383 |
| Low-dec vs high-persistent | 0.203 (0.046) | 4.376 (<.001) | 1.225 (0.057) | 1.118 | 1.341 |
| Mid-dec vs high-persistent | -0.031 (0.038) | -0.831 (.406) | 0.969 (0.037) | 0.900 | 1.043 |
|  |  |  |  |  |  |
| EA3_0.01 |  |  |  |  |  |
| Low-dec vs mid-dec | 0.060 (0.047) | 1.280 (.201) | 1.062 (0.050) | 0.969 | 1.164 |
| Low-dec vs high-persistent | 0.009 (0.048) | 0.181(.856) | 1.009 (0.048) | 0.919 | 1.108 |
| Mid-dec vs high-persistent | -0.051 (0.037) | -1.370 (.171) | 0.950 (0.036) | 0.883 | 1.022 |
|  |  |  |  |  |  |
| IQ_1 |  |  |  |  |  |
| Low-dec vs mid-dec | 0.200 (0.044) | 4.525 (<.001) | 1.221(0.054) | 1.120 | 1.331 |
| Low-dec vs high-persistent | 0.258 (0.047) | **5.515 (<.001)** | 1.295 (0.061) | 1.181 | 1.419 |
| Mid-dec vs high-persistent | 0.059 (0.039) | 1.491 (.136) | 1.060 (0.042) | 0.982 | 1.146 |
|  |  |  |  |  |  |
| IQ_0.3 |  |  |  |  |  |
| Low-dec vs mid-dec | 0.152 (0.044) | 3.433 (.001) | 1.165 (0.052) | 1.068 | 1.271 |
| Low-dec vs high-persistent | 0.218 (0.047) | 4.658 (<.001) | 1.244 (0.058) | 1.135 | 1.363 |
| Mid-dec vs high-persistent | 0.066 (0.039) | 1.669 (.095) | 1.068 (0.042) | 0.989 | 1.153 |
|  |  |  |  |  |  |
| IQ_0.01 |  |  |  |  |  |
| Low-dec vs mid-dec | 0.060 (0.044) | 1.367 (.172) | 1.062 (0.047) | 0.974 | 1.158 |
| Low-dec vs high-persistent | 0.093 (0.046) | 2.014 (.044) | 1.098 (0.051) | 1.002 | 1.202 |
| Mid-dec vs high-persistent | 0.033 (0.038) | 0.861 (.389) | 1.034 (0.040) | 0.959 | 1.114 |
|  |  |  |  |  |  |
| PSYCH_1 |  |  |  |  |  |
| Low-dec vs mid-dec | 0.009 (0.046) | 0.205 (.837) | 1.009 (0.046) | 0.923 | 1.104 |
| Low-dec vs high-persistent | 0.160 (0.047) | 3.392 (.001) | 1.173 (0.055) | 1.070 | 1.287 |
| Mid-dec vs high-persistent | 0.150 (0.038) | 3.934 (<.001) | 1.162 (0.045) | 1.078 | 1.253 |
|  |  |  |  |  |  |
| PSYCH_0.3 |  |  |  |  |  |
| Low-dec vs mid-dec | 0.011 (0.046) | 0.234 (.815) | 1.011 (0.045) | 1.079 | 1.254 |
| Low-dec vs high-persistent | 0.162 (0.047) | **3.426 (.001)** | 1.175 (0.055) | 1.072 | 1.289 |
| Mid-dec vs high-persistent | 0.151 (0.038) | 3.936 (<.001) | 1.163 (0.045) | 1.079 | 1.254 |
|  |  |  |  |  |  |
| PSYCH_0.01 |  |  |  |  |  |
| Low-dec vs mid-dec | 0.015 (0.044) | 0.343 (.731) | 1.015 (0.045) | 0.932 | 1.106 |
| Low-dec vs high-persistent | 0.119 (0.045) | 2.642 (.008) | 1.126 (0.051) | 1.031 | 1.229 |
| Mid-dec vs high-persistent | 0.104 (0.038) | 2.734 (.006) | 1.109 (0.042) | 1.030 | 1.195 |
|  |  |  |  |  |  |
| GP_1 |  |  |  |  |  |
| Low-dec vs mid-dec | -0.014 (0.049) | -0.284 (.777) | 0.986 (0.048) | 0.896 | 1.086 |
| Low-dec vs high-persistent | 0.182 (0.049) | 3.727 (<.001) | 1.200 (0.059) | 1.090 | 1.321 |
| Mid-dec vs high-persistent | 0.196 (0.038) | 5.232 (<.001) | 1.217 (0.046) | 1.131 | 1.310 |
|  |  |  |  |  |  |
| GP_0.3 |  |  |  |  |  |
| Low-dec vs mid-dec | -0.009 (0.049) | -0.189 (.850) | 0.991 (0.049) | 0.900 | 1.091 |
| Low-dec vs high-persistent | 0.186 (0.049) | **3.800 (<.001)** | 1.205 (0.059) | 1.094 | 1.326 |
| Mid-dec vs high-persistent | 0.196 (0.038) | 5.203 (<.001) | 1.216 (0.046) | 1.130 | 1.309 |
|  |  |  |  |  |  |
| GP_0.01 |  |  |  |  |  |
| Low-dec vs mid-dec | -0.016 (0.044) | -0.357 (.721) | 0.984 (0.043) | 0.903 | 1.073 |
| Low-dec vs high-persistent | 0.094 (0.046) | 2.060 (.039) | 1.099 (0.050) | 1.005 | 1.202 |
| Mid-dec vs high-persistent | 0.110 (0.039) | 2.831 (.005) | 1.116 (0.043) | 1.034 | 1.205 |
|  |  |  |  |  |  |
| SCZ_1 |  |  |  |  |  |
| Low-dec vs mid-dec | -0.052 (0.047) | -0.119 (.263) | 0.949 (0.044) | 0.866 | 1.040 |
| Low-dec vs high-persistent | -0.028 (0.047) | -0.592 (.554) | 0.972 (0.046) | 0.886 | 1.067 |
| Mid-dec vs high-persistent | 0.024 (0.038) | 0.641 (.522) | 1.025 (0.039) | 0.951 | 1.104 |
|  |  |  |  |  |  |
| SCZ_0.3 |  |  |  |  |  |
| Low-dec vs mid-dec | -0.032 (0.045) | -0.696 (.486) | 0.969 (0.044) | 0.886 | 1.059 |
| Low-dec vs high-persistent | -0.004 (0.047) | -0.082 (.935) | 0.996 (0.047) | 0.908 | 1.093 |
| Mid-dec vs high-persistent | 0.028 (0.039) | 0.719 (.472) | 1.028 (0.040) | 0.953 | 1.109 |
|  |  |  |  |  |  |
| SCZ_0.01 |  |  |  |  |  |
| Low-dec vs mid-dec | -0.069 (0.046) | -1.491 (.136) | 0.933 (0.043) | 0.852 | 1.022 |
| Low-dec vs high-persistent | -0.034 (0.047) | **-0.719 (.472)** | 0.967 (0.045) | 0.882 | 1.060 |
| Mid-dec vs high-persistent | 0.035 (0.038) | 0.934 (.350) | 1.036 (0.039) | 0.962 | 1.116 |
|  |  |  |  |  |  |
| OCD_1 |  |  |  |  |  |
| Low-dec vs mid-dec | 0.027 (0.045) | 0.600 (.548) | 1.027 (0.046) | 0.941 | 1.121 |
| Low-dec vs high-persistent | -0.008 (0.046) | -0.162 (.871) | 0.993 (0.046) | 0.906 | 1.087 |
| Mid-dec vs high-persistent | -0.034 (0.038) | -0.897 (.370) | 0.966 (0.037) | 0.896 | 1.042 |
|  |  |  |  |  |  |
| OCD_0.3 |  |  |  |  |  |
| Low-dec vs mid-dec | 0.028 (0.045) | 0.618 (.537) | 1.028 (0.046) | 0.942 | 1.122 |
| Low-dec vs high-persistent | -0.007 (0.046) | -0.157 (.875) | 0.993 (0.046) | 0.907 | 1.087 |
| Mid-dec vs high-persistent | -0.035 (0.038) | -0.911 (.362) | 0.966 (0.037) | 0.896 | 1.041 |
|  |  |  |  |  |  |
| OCD_0.01 |  |  |  |  |  |
| Low-dec vs mid-dec | 0.062 (0.045) | 1.372 (.170) | 1.064 (0.048) | 0.974 | 1.162 |
| Low-dec vs high-persistent | 0.010 (0.046) | **0.209 (.835)** | 1.010 (0.046) | 0.923 | 1.105 |
| Mid-dec vs high-persistent | 0.052 (0.038) | 1.372 (.170) | 0.949 (0.036) | 0.881 | 1.023 |
|  |  |  |  |  |  |
| MDD_1 |  |  |  |  |  |
| Low-dec vs mid-dec | 0.023 (0.044) | 0.518 (.604) | 1.023 (0.045) | 0.938 | 1.115 |
| Low-dec vs high-persistent | 0.202 (0.046) | 4.419 (<.001) | 1.224 (0.056) | 1.119 | 1.339 |
| Mid-dec vs high-persistent | 0.180 (0.039) | 4.613 (<.001) | 1.197 (0.047) | 1.109 | 1.292 |
|  |  |  |  |  |  |
| MDD_0.3 |  |  |  |  |  |
| Low-dec vs mid-dec | 0.026 (0.044) | 0.595 (.552) | 1.027 (0.045) | 0.942 | 1.119 |
| Low-dec vs high-persistent | 0.205 (0.046) | **4.475 (<.001)** | 1.224 (0.056) | 1.122 | 1.343 |
| Mid-dec vs high-persistent | 0.179 (0.039) | 4.599 (<.001) | 1.196 (0.047) | 1.108 | 1.291 |
|  |  |  |  |  |  |
| MDD_0.01 |  |  |  |  |  |
| Low-dec vs mid-dec | 0.077 (0.046) | 1.691 (.091) | 1.080 (0.049) | 0.988 | 1.181 |
| Low-dec vs high-persistent | 0.130 (0.046) | 2.811 (.005) | 1.139 (0.053) | 1.040 | 1.247 |
| Mid-dec vs high-persistent | 0.053 (0.038) | 1.406 (.160) | 1.055 (0.040) | 0.979 | 1.136 |
|  |  |  |  |  |  |
| BIP_1 |  |  |  |  |  |
| Low-dec vs mid-dec | -0.005 (0.046) | -0.103 (.918) | 0.995 (0.046) | 0.909 | 1.090 |
| Low-dec vs high-persistent | -0.022 (0.047) | **-0.476 (.634)** | 0.978 (0.046) | 0.892 | 1.072 |
| Mid-dec vs high-persistent | -0.017 (0.038) | -0.460 (.645) | 0.983 (0.037) | 0.912 | 1.059 |
|  |  |  |  |  |  |
| BIP_0.3 |  |  |  |  |  |
| Low-dec vs mid-dec | -0.005 (0.046) | -0.103 (.918) | 0.995 (0.046) | 0.909 | 1.090 |
| Low-dec vs high-persistent | -0.022 (0.047) | -0.464 (.643) | 0.979 (0.046) | 0.893 | 1.073 |
| Mid-dec vs high-persistent | -0.017 (0.038) | -0.446 (.656) | 0.983 (0.037) | 0.913 | 1.059 |
|  |  |  |  |  |  |
| BIP_0.01 |  |  |  |  |  |
| Low-dec vs mid-dec | 0.000 (0.046) | -0.004 (.997) | 1.000 (0.046) | 0.913 | 1.095 |
| Low-dec vs high-persistent | -0.018 (0.047) | -0.388 (.698) | 0.982 (0.046) | 0.896 | 1.076 |
| Mid-dec vs high-persistent | -0.018 (0.038) | -0.473 (.636) | 0.982 (0.037) | 0.912 | 1.058 |
|  |  |  |  |  |  |
| ASD_1 |  |  |  |  |  |
| Low-dec vs mid-dec | 0.162 (0.044) | 3.644 (<.001) | 1.176 (0.052) | 1.078 | 1.283 |
| Low-dec vs high-persistent | 0.253 (0.046) | 5.461 (<.001) | 1.287 (0.060) | 1.176 | 1.410 |
| Mid-dec vs high-persistent | 0.091 (0.038) | 2.361 (.018) | 1.095 (0.042) | 1.016 | 1.181 |
|  |  |  |  |  |  |
| ASD_0.3 |  |  |  |  |  |
| Low-dec vs mid-dec | 0.160 (0.044) | 3.599 (<.001) | 1.173 (0.052) | 1.076 | 1.280 |
| Low-dec vs high-persistent | 0.253 (0.046) | **5.464 (<.001)** | 1.288 (0.060) | 1.176 | 1.410 |
| Mid-dec vs high-persistent | 0.093 (0.038) | 2.417 (.016) | 1.097 (0.042) | 1.018 | 1.183 |
|  |  |  |  |  |  |
| ASD_0.01 |  |  |  |  |  |
| Low-dec vs mid-dec | 0.097 (0.045) | 2.160 (.031) | 1.101 (0.049) | 1.009 | 1.202 |
| Low-dec vs high-persistent | 0.207 (0.047) | 4.441 (<.001) | 1.230 (0.057) | 1.123 | 1.348 |
| Mid-dec vs high-persistent | 0.111 (0.038) | 2.879 (.004) | 1.117 (0.043) | 1.036 | 1.204 |
|  |  |  |  |  |  |
| ANOREX_1 |  |  |  |  |  |
| Low-dec vs mid-dec | 0.022 (0.046) | 0.478 (.633) | 1.022 (0.047) | 0.935 | 1.118 |
| Low-dec vs high-persistent | 0.079 (0.047) | 1.675 (.094) | 1.082 (0.051) | 0.987 | 1.186 |
| Mid-dec vs high-persistent | 0.057 (0.038) | 1.487 (.137) | 1.058 (0.040) | 0.982 | 1.140 |
|  |  |  |  |  |  |
| ANOREX_0.3 |  |  |  |  |  |
| Low-dec vs mid-dec | 0.022 (0.046) | 0.484 (.628) | 1.022 (0.047) | 0.935 | 1.118 |
| Low-dec vs high-persistent | 0.079 (0.047) | 1.683 (.092) | 1.082 (0.051) | 0.987 | 1.186 |
| Mid-dec vs high-persistent | 0.057 (0.038) | 1.490 (.136) | 1.058 (0.040) | 0.982 | 1.141 |
|  |  |  |  |  |  |
| ANOREX_0.01 |  |  |  |  |  |
| Low-dec vs mid-dec | 0.017 (0.046) | 0.366 (.714) | 1.017 (0.047) | 0.929 | 1.114 |
| Low-dec vs high-persistent | 0.083 (0.047) | **1.778 (.075)** | 1.087 (0.051) | 0.992 | 1.191 |
| Mid-dec vs high-persistent | 0.066 (0.038) | 1.736 (.082) | 1.068 (0.041) | 0.992 | 1.151 |
|  |  |  |  |  |  |
| ADHD_1 |  |  |  |  |  |
| Low-dec vs mid-dec | 0.041 (0.044) | 0.935 (.350) | 1.042 (0.046) | 0.956 | 1.136 |
| Low-dec vs high-persistent | 0.148 (0.046) | 3.211 (.001) | 1.159 (0.053) | 1.059 | 1.269 |
| Mid-dec vs high-persistent | 0.107 (0.039) | 2.766 (.006) | 1.113 (0.043) | 1.032 | 1.200 |
|  |  |  |  |  |  |
| ADHD_0.3 |  |  |  |  |  |
| Low-dec vs mid-dec | 0.044 (0.044) | 0.989 (.323) | 1.045 (0.046) | 0.958 | 1.139 |
| Low-dec vs high-persistent | 0.150 (0.046) | 3.259 (.001) | 1.162 (0.054) | 1.062 | 1.272 |
| Mid-dec vs high-persistent | 0.107 (0.039) | 2.763 (.006) | 1.112 (0.043) | 1.031 | 1.200 |
|  |  |  |  |  |  |
| ADHD_0.01 |  |  |  |  |  |
| Low-dec vs mid-dec | 0.082 (0.044) | 1.842 (.065) | 1.085 (0.048) | 0.995 | 1.184 |
| Low-dec vs high-persistent | 0.166 (0.046) | **3.602 (<.001)** | 1.181 (0.055) | 1.079 | 1.293 |
| Mid-dec vs high-persistent | 0.085 (0.039) | 2.181 (.029) | 1.088 (0.042) | 1.009 | 1.174 |

*Note. N =*7,090*.* Related and unrelated individuals included, using cluster-robust SE. The ‘low-decreasing’ class was used as the reference category. The ‘mid-decreasing’ class was used as the reference category for mid-dec vs high-persistent comparisons. GPS = genome-wide polygenic score (standardised). *f* = fraction of causal markers (at 1, 0.3, 0.01). *b* = unstandardized regression coefficient. Low-dec = low-decreasing class. Mid-dec = mid-decreasing class. High-persistent = high-persistent class. EA3 = years of education. IQ = intelligence. PSYCH = ever visited a psychiatrist for nerves, anxiety, tension, or depression. GP = ever visited a general practitioner for nerves, anxiety, tension, or depression. SCZ = schizophrenia. OCD = obsessive compulsive disorder. MDD = major depressive disorder. BIP = bipolar disorder. ASD = autism spectrum disorder. ANOREX = anorexia. ADHD = attention deficit hyperactivity disorder. Bold typeset indicates highest *z* statistic for the low-decreasing versus high-persistent comparison for each GPS.

**Supplementary Table 15.** Multinomial logistic regression results for paranoia latent trajectory class regressed on GPSs for most predictive *f*

|  | | **Single predictor regression** | | | | | **Multiple predictor regression** | | | | |
| --- | --- | --- | --- | --- | --- | --- | --- | --- | --- | --- | --- |
|  |  | **Beta** | | **Odds Ratio** | | | **Beta** | | **Odds Ratio** | | |
| **GPS variable** | ***f*** | ***b* (SE)** | **Z (*P* value)** | **OR (SE)** | **95% CI lower** | **95% CI upper** | ***b* (SE)** | **Z (*P* value)** | **OR (SE)** | **95% CI lower** | **95% CI upper** |
| **Years of education** | **1** |  | | | | |  | | | | |
| Low-dec vs mid-dec |  | 0.244 (0.045) | 5.389 (<.001)* | 1.277 (0.058) | 1.168 | 1.396 | 0.205 (0.051) | 4.056 (<.001)* | 1.228 (0.062) | 1.112 | 1.355 |
| Low-dec vs high-pers |  | 0.223 (0.046) | 4.816 (<.001)* | 1.250 (0.058) | 1.142 | 1.369 | 0.180 (0.052) | 3.478 (.001)* | 1.197 (0.062) | 1.082 | 1.325 |
| Mid-dec vs high-pers |  | -0.021 (0.038) | -0.550 (.583) | 0.979 (0.037) | 0.909 | 1.055 | -0.025 (0.043) | -0.579 (.562) | 0.975 (0.042) | 0.896 | 1.062 |
| **IQ** | **1** |  | | | | |  | | | | |
| Low-dec vs mid-dec |  | 0.200 (0.044) | 4.525 (<.001)* | 1.221(0.054) | 1.120 | 1.331 | 0.122 (0.49) | 2.522 (.012)* | 1.130 (0.055) | 1.028 | 1.243 |
| Low-dec vs high-pers |  | 0.258 (0.047) | 5.515 (<.001)* | 1.295 (0.061) | 1.181 | 1.419 | 0.214 (0.052) | 4.135 (<.001)* | 1.238 (0.064) | 1.119 | 1.370 |
| Mid-dec vs high-pers |  | 0.059 (0.039) | 1.491 (.136) | 1.060 (0.042) | 0.982 | 1.146 | 0.091 (0.044) | 2.090 (.037) | 1.095 (0.048) | 1.006 | 1.193 |
| **Psychiatrist** | **0.3** |  | | | | |  | | | | |
| Low-dec vs mid-dec |  | 0.011 (0.046) | 0.234 (.815) | 1.011 (0.045) | 1.079 | 1.254 | 0.019 (0.057) | 0.334 (.738) | 1.019 (0.058) | 0.912 | 1.139 |
| Low-dec vs high-pers |  | 0.162 (0.047) | 3.426 (.001)* | 1.175 (0.055) | 1.072 | 1.289 | 0.067 (0.060) | 1.117 (.264) | 1.069 (0.064) | 0.951 | 1.201 |
| Mid-dec vs high-pers |  | 0.151 (0.038) | 3.936 (<.001)* | 1.163 (0.045) | 1.079 | 1.254 | 0.048 (0.048) | 0.986 (.324) | 1.049 (0.051) | 0.954 | 1.153 |
| **GP** | **0.3** |  | | | | |  | | | | |
| Low-dec vs mid-dec |  | -0.009 (0.049) | -0.189 (.850) | 0.991 (0.049) | 0.900 | 1.091 | -0.006 (0.062) | -0.100 (.921) | 0.994 (0.062) | 0.880 | 1.123 |
| Low-dec vs high-pers |  | 0.186 (0.049) | 3.800 (<.001)* | 1.205 (0.059) | 1.094 | 1.326 | 0.117 (0.063) | 1.847 (.065) | 1.124 (0.071) | 0.993 | 1.273 |
| Mid-dec vs high-pers |  | 0.196 (0.038) | 5.203 (<.001)* | 1.216 (0.046) | 1.130 | 1.309 | 0.123 (0.049) | 2.524 (.012)* | 1.131 (0.055) | 1.028 | 1.245 |
| **Schizophrenia** | **0.01** |  | | | | |  | | | | |
| Low-dec vs mid-dec |  | -0.069 (0.046) | -1.491 (.136) | 0.933 (0.043) | 0.852 | 1.022 | - | - | - | - | - |
| Low-dec vs high-pers |  | -0.034 (0.047) | -0.719 (.472) | 0.967 (0.045) | 0.882 | 1.060 | - | - | - | - | - |
| Mid-dec vs high-pers |  | 0.035 (0.038) | 0.934 (.350) | 1.036 (0.039) | 0.962 | 1.116 | - | - | - | - | - |
| **OCD** | **0.01** |  | | | | |  | | | | |
| Low-dec vs mid-dec |  | 0.062 (0.045) | 1.372 (.170) | 1.064 (0.048) | 0.974 | 1.162 | - | - | - | - | - |
| Low-dec vs high-pers |  | 0.010 (0.046) | 0.209 (.835) | 1.010 (0.046) | 0.923 | 1.105 | - | - | - | - | - |
| Mid-dec vs high-pers |  | 0.052 (0.038) | 1.372 (.170) | 0.949 (0.036) | 0.881 | 1.023 | - | - | - | - | - |
| **MDD** | **0.3** |  | | | | |  | | | | |
| Low-dec vs mid-dec |  | 0.026 (0.044) | 0.595 (.552) | 1.027 (0.045) | 0.942 | 1.119 | 0.010 (0.049) | 0.198 (.843) | 1.010 (0.049) | 0.918 | 1.111 |
| Low-dec vs high-pers |  | 0.205 (0.046) | 4.475 (<.001)* | 1.228 (0.056) | 1.122 | 1.343 | 0.120 (0.52) | 2.331 (.020)* | 1.128 (0.058) | 1.019 | 1.248 |
| Mid-dec vs high-pers |  | 0.179 (0.039) | 4.599 (<.001)* | 1.196 (0.047) | 1.108 | 1.291 | 0.111 (0.043) | 2.548 (.011)* | 1.117 (0.048) | 1.026 | 1.216 |
| **Bipolar disorder** | **1** |  | | | | |  | | | | |
| Low-dec vs mid-dec |  | -0.005 (0.046) | -0.103 (.918) | 0.995 (0.046) | 0.909 | 1.090 | - | - | - | - | - |
| Low-dec vs high-pers |  | -0.022 (0.047) | -0.476 (.634) | 0.978 (0.046) | 0.892 | 1.072 | - | - | - | - | - |
| Mid-dec vs high-pers |  | -0.017 (0.038) | -0.460 (.645) | 0.983 (0.037) | 0.912 | 1.059 | - | - | - | - | - |
| **ASD** | **0.3** |  | | | | |  | | | | |
| Low-dec vs mid-dec |  | 0.160 (0.044) | 3.599 (<.001)* | 1.173 (0.052) | 1.076 | 1.280 | 0.104 (0.047) | 2.221 (.026) | 1.110 (0.052) | 1.012 | 1.217 |
| Low-dec vs high-pers |  | 0.253 (0.046) | 5.464 (<.001)* | 1.288 (0.060) | 1.176 | 1.410 | 0.141 (0.050) | 2.841 (.005)* | 1.151 (0.057) | 1.045 | 1.269 |
| Mid-dec vs high-pers |  | 0.093 (0.038) | 2.417 (.016)* | 1.097 (0.042) | 1.018 | 1.183 | 0.037 (0.041) | 0.889 (.374) | 1.037 (0.043) | 0.957 | 1.125 |
| **Anorexia** | **0.01** |  | | | | |  | | | | |
| Low-dec vs mid-dec |  | 0.017 (0.046) | 0.366 (.714) | 1.017 (0.047) | 0.929 | 1.114 | - | - | - | - | - |
| Low-dec vs high-pers |  | 0.083 (0.047) | 1.778 (.075) | 1.087 (0.051) | 0.992 | 1.191 | - | - | - | - | - |
| Mid-dec vs high-pers |  | 0.066 (0.038) | 1.736 (.082) | 1.068 (0.041) | 0.992 | 1.151 | - | - | - | - | - |
| **ADHD** | **0.01** |  | | | | |  | | | | |
| Low-dec vs mid-dec |  | 0.082 (0.044) | 1.842 (.065) | 1.085 (0.048) | 0.995 | 1.184 | 0.108 (0.047) | 2.288 (.022)* | 1.114 (0.053) | 1.016 | 1.222 |
| Low-dec vs high-pers |  | 0.166 (0.046) | 3.602 (<.001)* | 1.181 (0.055) | 1.079 | 1.293 | 0.157 (0.050) | 3.181 (.001)* | 1.171 (0.058) | 1.062 | 1.290 |
| Mid-dec vs high-pers |  | 0.085 (0.039) | 2.181 (.029) | 1.088 (0.042) | 1.009 | 1.174 | 0.050 (0.041) | 1.202 (.229) | 1.051 (0.043) | 0.969 | 1.139 |

*Note. N =*7,090*.* Related and unrelated individuals included, using cluster-robust SE. The ‘low-increasing’ class was used as the reference category. The ‘mid-decreasing’ class was used as the reference category for the mid-dec vs high-pers comparison. Results shown for the most predictive GPS *f* pertaining to the low-decreasing versus high-persistent comparison. GPS = genome-wide polygenic score (standardised). *f* = fraction of causal markers. *b* = unstandardized regression coefficient. Low-dec = low-decreasing class. Mid-dec = mid-decreasing class. High-pers = high-persistent class. IQ = intelligence. Psychiatrist = ever visited a psychiatrist for nerves, anxiety, tension, or depression. GP = ever visited a general practitioner for nerves, anxiety, tension, or depression. OCD = obsessive compulsive disorder. MDD = major depressive disorder. ASD = autism spectrum disorder. ADHD = attention deficit hyperactivity disorder. * = significant at *q* <.05 (FDR-adjusted *p* <.021 and *p* <.022 for single and multiple predictor regressions, respectively).

**Supplementary Table 16**. Multinomial logistic regression results for paranoia latent trajectory class regressed on family background variables

|  |  | **Single predictor regressions** | | | | | | | **Multiple predictor regression, *N* = 8942** | | | | |
| --- | --- | --- | --- | --- | --- | --- | --- | --- | --- | --- | --- | --- | --- |
| **Phenotypic variable** | ***N* for single predictor regressions** | **Beta** | | **Odds Ratio** | | | | | **Beta** | | **Odds Ratio** | | |
|  |  | ***b* (SE)** | **Z**  **(*P* value)** | **OR (SE)** | **95% CI lower** | | **95% CI upper** | | ***b* (SE)** | **Z**  **(*P* value)** | **OR (SE)** | **95% CI lower** | **95% CI upper** |
| **Male sex** | 12049 |  | | | | | | |  | | | | |
| Low-dec vs mid-dec |  | -0.163 (0.070) | -2.327 (.020)* | 0.849 (0.060) | 0.740 | | 0.975 | | -0.0271 (0.083) | -3.250 (.001)* | 0.763 (0.064) | 0.648 | 0.898 |
| Low-dec vs high-pers |  | -0.380 (0.074) | -5.161 (<.001)* | 0.684 (0.050) | 0.592 | | 0.790 | | -0.429 (0.087) | -4.927 (<.001)* | 0.651 (0.057) | 0.549 | 0.772 |
| Mid-dec vs high-pers |  | -0.216 (0.061) | -3.517 (<.001)* | 0.806 (0.050) | 0.714 | | 0.909 | | -0.159 (0.071) | -2.228 (.026)* | 0.853 (0.061) | 0.742 | 0.981 |
| **SES** | 11368 |  | | | | | | |  | | | | |
| Low-dec vs mid-dec |  | 0.319 (0.036) | 8.862 (<.001)* | 1.376 (0.050) | 1.282 | | 1.477 | | 0.344 (0.042) | 8.232 (<.001)* | 1.410 (0.059) | 1.299 | 1.531 |
| Low-dec vs high-pers |  | 0.221 (0.039) | 5.682 (<.001)* | 1.248 (0.049) | 1.156 | | 1.347 | | 0.267 (0.045) | 5.868 (<.001)* | 1.306 (0.059) | 1.194 | 1.427 |
| Mid-dec vs high-pers |  | -0.098 (0.033) | -3.007 (.003)* | 0.907 (0.030) | 0.851 | | 0.966 | | -0.077 (0.038) | -2.055 (.040) | 0.926 (0.035) | 0.860 | 0.996 |
| **Family history of schizophrenia** | 9673 |  | | | | | | |  | | | | |
| Low-dec vs mid-dec |  | 0.591 (0.282) | 2.093 (0.036)* | 1.805 (0.265) | | 1.038 | | 3.138 | 0.770 (0.346) | 2.226 (.026)* | 2.159 (0.746) | 1.096 | 4.251 |
| Low-dec vs high-pers |  | 0.981 (0.281) | 3.488 (<.001)* | 2.668 (0.751) | | 1.537 | | 4.632 | 1.102 (0.341) | 3.327 (.001)* | 3.011 (1.026) | 1.545 | 5.870 |
| Mid-dec vs high-pers |  | 0.391 (0.179) | 2.183 (0.029)* | 1.478 (0.265) | | 1.041 | | 2.100 | 0.333 (0.201) | 1.653 (.098) | 1.395 (0.281) | 0.940 | 2.070 |
| **Family history of bipolar disorder** | 9459 |  |  |  | |  | |  |  |  |  |  |  |
| Low-dec vs mid-dec |  | 0.196 (0.202) | 0.972 (.331) | 1.217 (0.245) | | 0.819 | | 1.806 | 0.012 (0.210) | 0.059 (.953) | 1.012 (0.213) | 0.671 | 1.529 |
| Low-dec vs high-pers |  | 0.478 (0.197) | 2.422 (.015)* | 1.613 (0.318) | | 1.095 | | 2.375 | 0.214 (0.208) | 1.026 (.305) | 1.238 (0.258) | 0.823 | 1.862 |
| Mid-dec vs high-pers |  | 0.282 (0.150) | 1.881 (.060) | 1.326 (0.199) | | 0.988 | | 1.779 | 0.201 (0.161) | 1.249 (.212) | 1.223 (0.197) | 0.892 | 1.677 |

*Note.* Related and unrelated individuals included, using cluster-robust SE. The ‘low-increasing’ class was used as the reference category. The ‘mid-decreasing’ class was used as the reference category for the mid-dec vs high-pers comparison. Low-dec = low-decreasing class. Mid-dec = mid-decreasing class. High-pers = high-persistent class. *b* = unstandardized regression coefficient. SES = socioeconomic status. * = significant at *q* <.05 (FDR-adjusted *p* <.042 and *p* <.029 for single and multiple predictor regressions, respectively).

**Supplementary Table 17**. Multinomial logistic regression results for paranoia latent trajectory class regressed on age 7 variables

|  |  | **Single predictor regressions** | | | | | **Multiple predictor regression, *N* = 7421** | | | | |
| --- | --- | --- | --- | --- | --- | --- | --- | --- | --- | --- | --- |
| **Phenotypic variable** | ***N* for single predictor regressions** | **Beta** | | **Odds Ratio** | | | **Beta** | | **Odds Ratio** | | |
|  |  | ***b* (SE)** | **Z**  **(*P* value)** | **OR (SE)** | **95% CI lower** | **95% CI upper** | ***b* (SE)** | **Z**  **(*P* value)** | **OR (SE)** | **95% CI lower** | **95% CI upper** |
| **Educational attainment** | 7662 |  | | | | |  | | | | |
| Low-dec vs mid-dec |  | 0.315 (0.047) | 6.768 (<.001)* | 1.371 (0.064) | 1.251 | 1.502 | 0.392 (0.051) | 7.650 (<.001)* | 1.480 (0.076) | 1.339 | 1.637 |
| Low-dec vs high-pers |  | 0.286 (0.049) | 5.867 (<.001)* | 1.331 (0.065) | 1.210 | 1.464 | 0.438 (0.053) | 8.203 (<.001)* | 1.550 (0.083) | 1.396 | 1.721 |
| Mid-dec vs high-pers |  | -0.030 (0.041) | -0.714 (.475) | 0.971 (0.040) | 0.895 | 1.053 | 0.046 (0.044) | 1.048 (.295) | 1.047 (0.046) | 0.961 | 1.140 |
| **Life events** | 9605 |  | | | | |  | | | | |
| Low-dec vs mid-dec |  | -0.014 (0.037) | -0.375 (.707) | 0.986 (0.036) | 0.918 | 1.060 | 0.001 (0.045) | 0.014 (.989) | 1.001 (0.045) | 0.917 | 1.092 |
| Low-dec vs high-pers |  | 0.083 (0.037) | 2.247 (.025)* | 1.086 (0.040) | 1.011 | 1.167 | 0.057 (0.045) | 1.276 (.202) | 1.059 (0.047) | 0.970 | 1.155 |
| Mid-dec vs high-pers |  | 0.096 (0.028) | 3.456 (.001)* | 1.101 (0.031) | 1.043 | 1.163 | 0.056 (0.032) | 1.774 (.076) | 1.058 (0.034) | 0.994 | 1.126 |
| **SDQ** | 9601 |  | | | | |  | | | | |
| Low-dec vs mid-dec |  | 0.014 (.009) | 1.529 (.126) | 1.014 0(.009) | 0.996 | 1.032 | 0.046 (0.011) | 4.041 (<.001)* | 1.047 (0.012) | 1.024 | 1.071 |
| Low-dec vs high-pers |  | 0.065 (.009) | 7.230 (<.001)* | 1.067 (0.010) | 1.049 | 1.086 | 0.097 (0.011) | 8.479 (<.001)* | 1.102 (0.013) | 1.077 | 1.127 |
| Mid-dec vs high-pers |  | 0.051 (0.007) | 7.545 (<.001)* | 1.053 (0.007) | 1.039 | 1.067 | 0.051 (0.008) | 6.357 (<.001)* | 1.052 (0.008) | 1.036 | 1.069 |

*Note.* Related and unrelated individuals included, using cluster-robust SE. The ‘low-increasing’ class was used as the reference category. The ‘mid-decreasing’ class was used as the reference category for the mid-dec vs high-pers comparison. Low-dec = low-decreasing class. Mid-dec = mid-decreasing class. High-pers = high-persistent class. *b* = unstandardized regression coefficient. SDQ = Strengths and Difficulties Questionnaire. * = significant at *q* <.05 (FDR-adjusted *p* <.033 and *p* <.028 for single and multiple predictor regressions, respectively).

**Supplementary Table 18.** Longitudinal measurement analysis results for hallucinations

|  | Parameters | Fit indices | | | Comparison of fit indices between nested models | | |
| --- | --- | --- | --- | --- | --- | --- | --- |
|  |  | CFI | RMSEA [90% CI] | SRMR | Δ CFI | Δ RMSEA | Δ SRMR |
| Configural invariance model (no constraints) | 144 | 0.978 | 0.016 [0.015, 0.018] | 0.025 | - | - | - |
| Metric invariance model (factor loadings constrained) | 132 | 0.978 | 0.016 [0.014, 0.017] | 0.028 | 0.000 | 0.000 | -0.003 |
| Scalar invariance model (factor loadings and intercepts constrained) | 120 | 0.965 | 0.020 [0.018, 0.021] | 0.033 | 0.013 | -0.004 | -0.005 |
| Partial scalar invariance model (factor loadings and intercepts constrained) ^a^ | 122 | 0.974 | 0.017 [0.015, 0.018] | 0.029 | 0.004 ^b^ | -0.001 ^b^ | -0.001 ^b^ |

*Note. N =* 6,032 (one randomly selected twin per pair). CFI = comparative fit index. RMSEA = root mean square error of approximation. SRMR = standardized root mean square residual. Δ denotes change value. ^a^ Item 1 intercepts free to vary. ^b^ Change values compared to metric invariance model. The partial strict model resulted in non-positive definite latent variable matrix so the results are not reported. The measurement model was a 3-factor model (age 16: χ^2^ (24) = 137.68, *p* <.001, CFI = 0.98, RMSEA = 0.05 [0.04, 0.06], SRMR = 0.02; age 17: χ^2^ (24) = 65.70, *p* <.001, CFI = 0.99, RMSEA = 0.05 [0.04, 0.06], SRMR = 0.02; age 22: χ^2^ (24) = 108.52, *p* <.001, CFI = 0.98, RMSEA = 0.06 [0.05, 0.08], SRMR = 0.02). Confirmatory factor analysis results available in full from corresponding author.

**Supplementary Table 19.** Latent growth curve model fit results for results for hallucinations

|  | Par. | LL | AIC | BIC | χ^2^ value (*df*) | CFI | RMSEA [90% CI] | SRMR |
| --- | --- | --- | --- | --- | --- | --- | --- | --- |
| Intercept only model  **Linear growth model** ^a^  Quadratic growth model ^b^  Latent basis growth model ^c^ | 5  **8**  8  7 | -66359.938  **-64621.298**  -64541.081  -64571.621 | 132729.876**129258.596**  129098.162  129159.243 | 132766.862**129317.775**  129157.340  129218.421 | 1581.152 (4), *p* <.001  **189.755 (1), *p* <.001**  2.193 (1), *p* <.001  20.890 (1), *p* <.001 | 0.000  **0.933**  0.999  0.971 | 0.274 [0.263, 0.286]  **0.117 [0.101, 0.131]**  0.015 [0.000, 0.043]  0.071 [0.051, 0.101] | 0.247  **0.070**  0.012  0.041 |

*Note. N =* 12,056. Related and unrelated individuals included, using cluster-robust SE. LL = loglikelihood value. AIC = Akaike’s Information Criterion. BIC = Bayesian Information Criterion. χ^2^ = chi-square value. CFI = comparative fit index. RMSEA = root mean square error of approximation. SRMR = standardized root mean square residual. ^a^ Residual variances were freely estimated at each time-point. ^b^ Quadratic slope variance-covariance parameters not estimated, and residual variances constrained to equality (to achieve identification and over-identification, respectively). Removal of the age 22 residual variance was required to obtain a proper solution for the over-identified model. ^c^ A proper solution could not be obtained for an over-identified latent basis model (with residual variances constrained to equality). Removal of the age 22 residual variance was required to obtain a proper solution of the over-identified model. Bold typeset indicates model that was selected for subsequent growth mixture modelling.

**Supplementary Table 20.** Parameter estimates from linear growth curve model of hallucinations

|  | Mean | | | | | |  | |  | |  | | Variance |  |  |
| --- | --- | --- | --- | --- | --- | --- | --- | --- | --- | --- | --- | --- | --- | --- | --- |
|  | Estimate | | SE | | *z* | | *p* | |  | | Estimate | | SE | *z* | *p* |
| Intercept  Slope  16 years  17 years  22 years | 4.88  -0.53  -  -  - | | 0.07  0.01  -  -  - | | 67.16  -41.96  -  -  - | | <.001  <.001  -  -  - | |  | | 27.98  0.60  9.85  28.21  5.03 | | 1.23  0.14  1.13  1.62  4.24 | 23.126  19.368  8.70  17.44  1.19 | <.001  <.001  <.001  <.001  0.24 |
|  | | Estimate | | SE | | *z* | | *p* | | Standardised  estimate | |  |  |  |  |
| Factor covariance | | -3.13 | | 0.21 | | -15.26 | | <.001 | | -0.76 | |  |  |  |  |

*Note. N =* 12,056. Related and unrelated individuals included, using cluster-robust SE. Unstandardised estimates (unless otherwise indicated).

**Supplementary Table 21.** Growth mixture model fit results for hallucinations

| *k* | Model | Par. | Constraints | LL | BIC | AIC | Entropy ^1^ |
| --- | --- | --- | --- | --- | --- | --- | --- |
| 1  1  2  2  2  2  2  2  2  2  3  3  3  3  3  3  3  3 | Model LCGA  Model 0  Model LCGA  Model 0  Model 1A  Model 1B  Model 1C  Model 2A  Model 2B  Model 2C  Model LCGA  Model 0  Model 1A  Model 1B  Model 1C  Model 2A  Model 2B  Model 2C | 5  8  11  17  13  13  15  12  11  12  17  26  20  20  22  18  15  16 | No growth factor variances  None  No growth factor variances  None  Within-class residual variances  Between-class residual variances  Between-class growth factor variances  Within-class and between-class residual variances  Within-class residual variances and between-class growth factor variances  Between-class residual variances and between-class growth factor variances  No growth factor variances  None  Within-class residual variances  Between-class residual variances  Between-class growth factor variances  Within-class and between-class residual variances  Within-class residual variances and between-class growth factor variances  Between-class residual variances and between-class growth factor variances | -65917.295  -64673.683  -  -  -53996.157  -  -  -60064.952  -54440.670  -  -  -  -  -  -  -59209.341  -  - | 131881.575  **129422.544**  -  -  **108114.477**  -  -  120242.670  108984.709  -  -  -  -  -  -  **118587.830**  -  - | 131844.589  129363.367  -  -  108018.314  -  -  120153.904  108903.340  -  -  -  -  -  -  118454.682  -  - | -  -  -  -  0.776  -  -  0.744  0.779  -  -  -  -  -  -  0.768  -  - |
|  |  |  |  |  |  |  |  |

*Note. k* = number of classes. Par. = number of estimated parameters (for final model if converged, for unadjusted model if not converged). LL = loglikelihood value. AIC = Akaike’s Information Criterion. BIC = Bayesian Information Criterion. ^1^ = No calculation for 1-class model. Bold typeset indicates lowest BIC value for each *k*-class model. Loglikelihood values replicated for best-fitting *k-*class models using the two random seed values with the highest loglikelihoods.

**Supplementary Table 22.** Parameter estimates for each best fitting *k-*class model for hallucinations

| *k* | Model | Parameter | Class 1  Mean (SE) | *P* | Variance (SE) | *P* | Class 2  Mean (SE) | *P* | Variance (SE) | *P* | Class 3  Mean (SE) | *P* | Variance (SE) | *P* |
| --- | --- | --- | --- | --- | --- | --- | --- | --- | --- | --- | --- | --- | --- | --- |
| 1 | Model 0 | Intercept  Linear slope  W1  W2  W3  Covariance | 5.031 (0.074)  -0.475 (0.011)  -  -  -  - | <.001  <.001  -  -  -  - | 26.878 (1.241)  0.253 (0.090)  12.799 (1.092)  26.480 (1.558)  12.101 (3.472)  -2.437 (0.184) | <.001  .005  <.001  <.001  <.001  <.001 | -  -  -  -  -  - | -  -  -  -  -  - | -  -  -  -  -  - | -  -  -  -  -  - | -  -  -  -  -  - | -  -  -  -  -  - | -  -  -  -  -  - | -  -  -  -  -  - |
| 2 | Model 1A | Intercept  Linear slope  W1  W2  W3  Covariance | 8.828 (0.139)  -0.754 (0.20)  -  -  -  - | <.001  <.001  -  -  -  - | 25.651 (1.603)  0.237 (0.072)  27.066 (1.332)  27.066 (1.332)  27.066 (1.332)  -2.385 (0.261) | <.001  <.001  <.001  <.001  <.001  <.001 | 1.209 (0.055)  -0.158 (0.007)  -  -  -  - | <.001  <.001  -  -  -  - | 1.736 (0.135)  0.034 (0.002)  0.189 (0.015)  0.189 (0.015)  0.189 (0.015)  -0.244 (0.017) | <.001  <.001  <.001  <.001  <.001  <.001 | -  -  -  -  -  - | -  -  -  -  -  - | -  -  -  -  -  - | -  -  -  -  -  - |
| 3 | Model 2A | Intercept  Linear slope  W1  W2  W3  Covariance | 2.050 (0.125)  -0.206 (0.016)  -  -  -  - | <.001  <.001  -  -  -  - | 0.025 (0.519)  0.001 (0.022)  5.846 (0.214)  5.486 (0.214)  5.486 (0.214)  -0.003 (0.100) | .962  .980  <.001  <.001  <.001  .973 | 10.917 (0.425)  -1.117 (0.033)  -  -  -  - | <.001  <.001  -  -  -  - | 24.983 (2.494)  0.885 (0.145)  5.846 (0.214)  5.486 (0.214)  5.486 (0.214)  -4.011 (0.477) | <.001  <.001  <.001  <.001  <.001  <.001 | 18.514 (1.073)  0.325 (0.614)  -  -  -  - | <.001  .596  -  -  -  - | 218.790 (69.883)  109.451 (54.558)  5.846 (0.214)  5.486 (0.214)  5.486 (0.214)  -103.138 (46.195) | <.001  .045  <.001  <.001  <.001  .026 |

*Note. k* = number of classes. W1-W3 = data collection waves 1-3. Variance of W1-W3 represents residual variance at data collection waves 1-3. Model 0: Unconstrained model. Model 1A: Model with within-class residual variances constrained. Model 2A: Model with within-class and between-class residual variances constrained.

**Supplementary Table 23.** Most likely class classification values for each best fitting *k-*class model for hallucinations

| *k* | Model |  | Classification probabilities | | | Final class counts and proportions |
| --- | --- | --- | --- | --- | --- | --- |
|  |  |  | Class 1 | Class 2 | Class 3 |  |
| 1 | Model 0 | Class 1 | 1.000 | - | - | 12054 (100%) |
| 2 | Model 1A | Class 1  Class 2 | 0.972  0.090 | 0.028  0.910 | -  - | 6610 (54.84%)  5444 (45.16%) |
| 3 | Model 2A | Class 1  Class 2  Class 3 | 0.978  0.241  0.046 | 0.022  0.748  0.242 | 0.000  0.011  0.712 | 9142 (75.84%)  2563 (21.26%)  349 (2.90%) |

*Note. k* = number of classes. Model 0: Unconstrained model. Model 1A: Model with within-class residual variances constrained. Model 2A: Model with within-class and between-class residual variances constrained. Values based on most likely latent class membership.

**Supplementary Table 24**. Multinomial logistic regression results for hallucinations latent trajectory class regressed on GPSs for all GPS *f*

|  | Beta | | Odds Ratio | | |
| --- | --- | --- | --- | --- | --- |
|  | *b* (SE) | Z (P value) | OR (SE) | 95% CI lower bound | 95% CI upper bound |
| EA3_1 |  |  |  |  |  |
| Low-decreasing vs mid-decreasing | -0.074 (0.028) | **-2.648 (.008)** | 0.929 (0.026) | 0.880 | 0.981 |
|  |  |  |  |  |  |
| EA3_0.3 |  |  |  |  |  |
| Low-decreasing vs mid-decreasing | -0.055 (0.028) | -1.999 (.046) | 0.946 (0.026) | 0.896 | 0.999 |
|  |  |  |  |  |  |
| EA3_0.01 |  |  |  |  |  |
| Low-decreasing vs mid-decreasing | -0.018 (0.028) | -0.645 (.519) | 0.982 (0.028) | 0.982 | 1.038 |
|  |  |  |  |  |  |
| IQ_1 |  |  |  |  |  |
| Low-decreasing vs mid-decreasing | -0.013 (0.028) | -0.455 (.649) | 0.987 (0.027) | 0.935 | 1.043 |
|  |  |  |  |  |  |
| IQ_0.3 |  |  |  |  |  |
| Low-decreasing vs mid-decreasing | -0.005 (0.028) | -0.194 (.846) | 0.995 (0.028) | 0.942 | 1.050 |
|  |  |  |  |  |  |
| IQ_0.01 |  |  |  |  |  |
| Low-decreasing vs mid-decreasing | 0.025 (0.028) | **0.892 (.373)** | 1.025 (0.028) | 0.971 | 1.082 |
|  |  |  |  |  |  |
| PSYCH_1 |  |  |  |  |  |
| Low-decreasing vs mid-decreasing | 0.090 (0.028) | 3.245 (.001) | 1.094 (0.030) | 1.036 | 1.155 |
|  |  |  |  |  |  |
| PSYCH_0.3 |  |  |  |  |  |
| Low-decreasing vs mid-decreasing | 0.091 (0.028) | **3.288 (.001)** | 1.095 (0.030) | 1.037 | 1.156 |
|  |  |  |  |  |  |
| PSYCH_0.01 |  |  |  |  |  |
| Low-decreasing vs mid-decreasing | 0.053 (0.028) | 1.915 (.056) | 1.054 (0.029) | 0.999 | 1.113 |
|  |  |  |  |  |  |
| GP_1 |  |  |  |  |  |
| Low-decreasing vs mid-decreasing | 0.117 (0.028) | **4.220 (<.001)** | 1.124 (0.031) | 1.065 | 1.187 |
|  |  |  |  |  |  |
| GP_0.3 |  |  |  |  |  |
| Low-decreasing vs mid-decreasing | 0.117 (0.028) | 4.216 (<.001) | 1.124 (0.031) | 1.065 | 1.187 |
|  |  |  |  |  |  |
| GP_0.01 |  |  |  |  |  |
| Low-decreasing vs mid-decreasing | 0.044 (0.028) | 1.613 (.107) | 1.045 (0.029) | 0.990 | 1.103 |
|  |  |  |  |  |  |
| SCZ_1 |  |  |  |  |  |
| Low-decreasing vs mid-decreasing | -0.019 (0.028) | **-0.698 (.485)** | 0.981 (0.027) | 0.929 | 1.036 |
|  |  |  |  |  |  |
| SCZ_0.3 |  |  |  |  |  |
| Low-decreasing vs mid-decreasing | -0.013 (0.028) | -0.453 (.650) | 0.988 (0.027) | 0.935 | 1.043 |
|  |  |  |  |  |  |
| SCZ_0.01 |  |  |  |  |  |
| Low-decreasing vs mid-decreasing | -0.004 (0.028) | -0.145 (.884) | 0.996 (0.027) | 0.944 | 1.051 |
|  |  |  |  |  |  |
| OCD_1 |  |  |  |  |  |
| Low-decreasing vs mid-decreasing | 0.025 (0.027) | 0.927 (.354) | 1.026 (0.028) | 0.972 | 1.082 |
|  |  |  |  |  |  |
| OCD_0.3 |  |  |  |  |  |
| Low-decreasing vs mid-decreasing | 0.025 (0.027) | 0.929 (.353) | 1.026 (0.028) | 0.972 | 1.082 |
|  |  |  |  |  |  |
| OCD_0.01 |  |  |  |  |  |
| Low-decreasing vs mid-decreasing | 0.028 (0.027) | **1.028 (.304)** | 1.029 (0.028) | 0.975 | 1.085 |
|  |  |  |  |  |  |
| MDD_1 |  |  |  |  |  |
| Low-decreasing vs mid-decreasing | 0.063 (0.027) | **2.284 (.022)** | 1.065 (0.029) | 1.009 | 1.124 |
|  |  |  |  |  |  |
| MDD_0.3 |  |  |  |  |  |
| Low-decreasing vs mid-decreasing | 0.062 (0.027) | 2.273 (.023) | 1.064 (0.029) | 1.009 | 1.123 |
|  |  |  |  |  |  |
| MDD_0.01 |  |  |  |  |  |
| Low-decreasing vs mid-decreasing | -0.003 (0.027) | -0.097 (.922) | 0.997 (0.027) | 0.945 | 1.052 |
|  |  |  |  |  |  |
| BIP_1 |  |  |  |  |  |
| Low-decreasing vs mid-decreasing | -0.033 (0.028) | -1.183 (.237) | 0.968 (0.027) | 0.916 | 1.022 |
|  |  |  |  |  |  |
| BIP_0.3 |  |  |  |  |  |
| Low-decreasing vs mid-decreasing | -0.034 (0.028) | -1.204 (.228) | 0.967 (0.027) | 0.915 | 1.021 |
|  |  |  |  |  |  |
| BIP_0.01 |  |  |  |  |  |
| Low-decreasing vs mid-decreasing | -0.039 (0.028) | **-1.409 (.159)** | 0.961 (0.027) | 0.910 | 1.015 |
|  |  |  |  |  |  |
| ASD_1 |  |  |  |  |  |
| Low-decreasing vs mid-decreasing | 0.099 (0.028) | 3.552 (<.001) | 1.104 (0.031) | 1.045 | 1.166 |
|  |  |  |  |  |  |
| ASD_0.3 |  |  |  |  |  |
| Low-decreasing vs mid-decreasing | 0.099 (0.028) | **3.557 (<.001)** | 1.104 (0.031) | 1.045 | 1.166 |
|  |  |  |  |  |  |
| ASD_0.01 |  |  |  |  |  |
| Low-decreasing vs mid-decreasing | 0.089 (0.028) | 3.222 (.001) | 1.093 (0.030) | 1.035 | 1.154 |
|  |  |  |  |  |  |
| ANOREX_1 |  |  |  |  |  |
| Low-decreasing vs mid-decreasing | -0.005 (0.027) | -0.181 (.856) | 0.995 (0.027) | 0.943 | 1.050 |
|  |  |  |  |  |  |
| ANOREX_0.3 |  |  |  |  |  |
| Low-decreasing vs mid-decreasing | -0.006 (0.027) | -0.204 (.839) | 0.994 (0.027) | 0.942 | 1.049 |
|  |  |  |  |  |  |
| ANOREX_0.01 |  |  |  |  |  |
| Low-decreasing vs mid-decreasing | -0.010 (0.028) | **-0.368 (.713)** | 0.990 (0.027) | 0.938 | 1.045 |
|  |  |  |  |  |  |
| ADHD_1 |  |  |  |  |  |
| Low-decreasing vs mid-decreasing | 0.084 (0.028) | 3.055 (.002) | 1.088 (0.030) | 1.031 | 1.148 |
|  |  |  |  |  |  |
| ADHD_0.3 |  |  |  |  |  |
| Low-decreasing vs mid-decreasing | 0.085 (0.028) | **3.080 (.002)** | 1.088 (0.030) | 1.031 | 1.149 |
|  |  |  |  |  |  |
| ADHD_0.01 |  |  |  |  |  |
| Low-decreasing vs mid-decreasing | 0.063 (0.027) | 2.303 (.021) | 1.065 (0.029) | 1.009 | 1.124 |

*Note. N =*7,093*.* Related and unrelated individuals included, using cluster-robust SE. The ‘low-decreasing’ class was used as the reference category. GPS = genome-wide polygenic score (standardised). *f* = fraction of causal markers (at 1, 0.3, 0.01). *b* = unstandardized regression coefficient. Low-decreasing = low-decreasing class. Mid-decreasing = mid-decreasing class. EA3 = years of education. IQ = intelligence. PSYCH = ever visited a psychiatrist for nerves, anxiety, tension, or depression. GP = ever visited a general practitioner for nerves, anxiety, tension, or depression. SCZ = schizophrenia. OCD = obsessive compulsive disorder. MDD = major depressive disorder. BIP = bipolar disorder. ASD = autism spectrum disorder. ANOREX = anorexia. ADHD = attention deficit hyperactivity disorder. Bold typeset indicates highest *z* statistic for each GPS.

**Supplementary Table 25**. Multinomial logistic regression results for hallucinations latent trajectory class regressed on GPSs for most predictive *f*

|  |  | **Single predictor regressions** | | | | | **Multiple predictor regression** | | | | |
| --- | --- | --- | --- | --- | --- | --- | --- | --- | --- | --- | --- |
|  |  | **Beta** | | **Odds Ratio** | | | **Beta** | | **Odds Ratio** | | |
| **GPS** | ***f*** | ***b* (SE)** | **Z (*P* value)** | **OR (SE)** | **95% CI lower** | **95% CI upper** | ***b* (SE)** | **Z (*P* value)** | **OR (SE)** | **95% CI lower** | **95% CI upper** |
| **Years of education** | **1** |  | | | | |  | | | | |
| Low-dec vs mid-dec |  | -0.074 (0.028) | -2.648 (.008)* | 0.929 (0.026) | 0.880 | 0.981 | -0.063 (0.029) | -2.153 (.031) | 0.939 (0.027) | 0.887 | 0.994 |
| **IQ** | **0.01** |  | | | | |  | | | | |
| Low-dec vs mid-dec |  | 0.025 (0.028) | 0.892 (.373) | 1.025 (0.028) | 0.971 | 1.082 | - | - | - | - | - |
| **Visited a psychiatrist** | **0.3** |  | | | | |  | | | | |
| Low-dec vs mid-dec |  | 0.091 (0.028) | 3.288 (.001)* | 1.095 (0.030) | 1.037 | 1.156 | 0.032 (0.035) | 0.922 (.357) | 1.033 (0.036) | 0.965 | 1.105 |
| **Visited a GP** | **1** |  | | | | |  | | | | |
| Low-dec vs mid-dec |  | 0.117 (0.028) | 4.220 (<.001)* | 1.124 (0.031) | 1.065 | 1.187 | 0.083 (0.036) | 2.314 (.021) | 1.087 (0.039) | 1.013 | 1.166 |
| **Schizophrenia** | **1** |  | | | | |  | | | | |
| Low-dec vs mid-dec |  | -0.019 (0.028) | -0.698 (.485) | 0.981 (0.027) | 0.929 | 1.036 | - | - | - | - | - |
| **OCD** | **0.01** |  | | | | |  | | | | |
| Low-dec vs mid-dec |  | 0.028 (0.027) | 1.028 (.304) | 1.029 (0.028) | 0.975 | 1.085 | - | - | - | - | - |
| **MDD** | **1** |  | | | | |  | | | | |
| Low-dec vs mid-dec |  | 0.063 (0.027) | 2.284 (.022)* | 1.065 (0.029) | 1.009 | 1.124 | -0.010 (0.031) | -0.324 (.746) | 0.990 (0.030) | 0.932 | 1.052 |
| **Bipolar disorder** | **0.01** |  | | | | |  | | | | |
| Low-dec vs mid-dec |  | -0.039 (0.028) | -1.409 (.159) | 0.961 (0.027) | 0.910 | 1.015 | - | - | - | - | - |
| **ASD** | **0.3** |  | | | | |  | | | | |
| Low-dec vs mid-dec |  | 0.099 (0.028) | 3.557 (<.001)* | 1.104 (0.031) | 1.045 | 1.166 | 0.084 (0.030) | 2.820 (.005)* | 1.088 (0.033) | 1.026 | 1.154 |
| **Anorexia** | **0.01** |  | | | | |  | | | | |
| Low-dec vs mid-dec |  | -0.010 (0.028) | -0.368 (.713) | 0.990 (0.027) | 0.938 | 1.045 | - | - | - | - | - |
| **ADHD** | **0.3** |  | | | | |  | | | | |
| Low-dec vs mid-dec |  | 0.085 (0.028) | 3.080 (.002)* | 1.088 (0.030) | 1.031 | 1.149 | 0.040 (0.030) | 1.317 (.188) | 1.041(0.031) | 0.981 | 1.104 |

*Note. N =*7,093*.* Related and unrelated individuals included, using cluster-robust SE. The ‘low-decreasing’ class was used as the reference category. GPS = genome-wide polygenic score (standardised). *f* = fraction of causal markers. *b* = unstandardized regression coefficient. Low-dec = low-decreasing class. Mid-dec = mid-decreasing class. IQ = intelligence. Visited a psychiatrist = ever visited a psychiatrist for nerves, anxiety, tension, or depression. Visited a GP = ever visited a general practitioner for nerves, anxiety, tension, or depression. OCD = obsessive compulsive disorder. MDD = major depressive disorder. ASD = autism spectrum disorder. ADHD = attention deficit hyperactivity disorder. * = significant at *q* <.05 (FDR-adjusted *p* <.027 and *p* <.008 for single and multiple predictor regressions, respectively).

**Supplementary Table 26**. Multinomial logistic regression results for hallucinations latent trajectory class regressed on family background variables

|  |  | **Single predictor regressions** | | | | | **Multiple predictor regression, *N* = 8946** | | | | |
| --- | --- | --- | --- | --- | --- | --- | --- | --- | --- | --- | --- |
| **Phenotypic variable** | ***N* for single predictor regressions** | **Beta** | | **Odds Ratio** | | | **Beta** | | **Odds Ratio** | | |
|  |  | ***b* (SE)** | **Z**  **(*P* value)** | **OR (SE)** | **95% CI lower** | **95% CI upper** | ***b* (SE)** | **Z**  **(*P* value)** | **OR (SE)** | **95% CI lower** | **95% CI upper** |
| **Male sex** |  |  | | | | |  | | | | |
| Low-dec vs mid-dec | 12054 | -0.168 (0.044) | -3.807 (<.001)* | 0.846 (0.037) | 0.776 | 0.922 | -0.241 (0.051) | -4.715 (<.001)* | 0.786 (0.040) | 0.711 | 0.868 |
| **SES** |  |  | | | | |  | | | | |
| Low-dec vs mid-dec | 11373 | -0.093 (0.024) | -3.912 (<.001)* | 0.911 (0.022) | 0.870 | 0.955 | -0.082 (0.027) | -3.006 (.003)* | 0.92 (0.025) | 0.873 | 0.972 |
| **Family history of schizophrenia** |  |  | | | | |  | | | | |
| Low-dec vs mid-dec | 9678 | 0.268 (0.130) | 2.066 (.039)* | 1.307 (0.169) | 1.014 | 1.684 | 0.332 (0.147) | 2.263 (.024)* | 1.394 (0.205) | 1.046 | 1.860 |
| **Family history of bipolar disorder** |  |  | | | | |  | | | | |
| Low-dec vs mid-dec | 9463 | 0.27 (0.109) | 2.497 (.013)* | 1.311 (0.142) | 1.060 | 1.622 | 0.25 (0.118) | 2.135 (.033)* | 1.286 (0.151) | 1.021 | 1.619 |

*Note.* Related and unrelated individuals included, using cluster-robust SE. The ‘low-decreasing’ class was used as the reference category. *b* = unstandardized regression coefficient. SES = socioeconomic status. * = significant at *q* <.05 (FDR-adjusted *p* <.05 and *p* <.05 for single and multiple predictor regressions, respectively).

**Supplementary Table 27**. Multinomial logistic regression results for hallucinations latent trajectory class regressed on age 7 variables

|  |  | **Single predictor regressions** | | | | | **Multiple predictor regression, *N* = 7424** | | | | |
| --- | --- | --- | --- | --- | --- | --- | --- | --- | --- | --- | --- |
| **Phenotypic variable** | ***N* for single predictor regressions** | **Beta** | | **Odds Ratio** | | | **Beta** | | **Odds Ratio** | | |
|  |  | ***b* (SE)** | **Z**  **(*P* value)** | **OR (SE)** | **95% CI lower** | **95% CI upper** | ***b* (SE)** | **Z (*P* value)** | **OR (SE)** | **95% CI lower** | **95% CI upper** |
| **Educational attainment** |  |  | | | | |  | | | | |
| Low-dec vs mid-dec | 7665 | -0.116 (0.029) | -3.954 (<.001)* | 0.890 (0.026) | 0.840 | 0.943 | -0.063 (0.031) | -2.051 (.040) | 0.939 (0.029) | 0.883 | 0.997 |
| **Life events** |  |  | | | | |  | | | | |
| Low-dec vs mid-dec | 9611 | 0.060 (0.021) | 2.878 (.004)* | 1.062 (0.022) | 1.019 | 1.107 | 0.029 (0.024) | 1.233 (.218) | 1.030 (0.025) | 0.983 | 1.079 |
| **SDQ** |  |  | | | | |  | | | | |
| Low-dec vs mid-dec | 9607 | 0.042 (0.005) | 8.023 (<.001)* | 1.043 (0.005) | 1.032 | 1.054 | 0.040 (0.006) | 6.443 (<.001)* | 1.040 (0.006) | 1.028 | 1.053 |

*Note.* Related and unrelated individuals included, using cluster-robust SE. The ‘low-decreasing’ class was used as the reference category. *b* = unstandardized regression coefficient. SDQ = Strengths and Difficulties Questionnaire. * = significant at *q* <.05 (FDR-adjusted *p* <.05 and *p* <.017 for single and multiple predictor regressions, respectively).

**Supplementary Table 28.** Longitudinal measurement invariance analysis results for negative symptoms

|  | Parameters | Fit indices | | | Comparison of fit indices between nested models | | |
| --- | --- | --- | --- | --- | --- | --- | --- |
|  |  | CFI | RMSEA [90% CI] | SRMR | Δ CFI | Δ RMSEA | Δ SRMR |
| Configural invariance model (no constraints) | 189 | 0.992 | 0.014 [0.012, 0.016] | 0.016 | - | - | - |
| Metric invariance model (factor loadings constrained) | 183 | 0.991 | 0.014 [0.012, 0.016] | 0.018 | 0.001 | 0.000 | 0.002 |
| Scalar invariance model (factor loadings and intercepts constrained) | 177 | 0.988 | 0.016 [0.014, 0.018] | 0.020 | 0.003 | -0.002 | -0.002 |
| Strict invariance model (factor loadings, intercepts and residual variances constrained) | 165 | 0.968 | 0.025 [0.023, 0.027] | 0.030 | 0.020 | -0.009 | -0.010 |
| Partial strict invariance model (factor loadings, intercepts and residual variances constrained, excluding item 2) ^a^ | 171 | 0.980 | 0.020 [0.019, 0.022] | 0.026 | 0.008 ^b^ | -0.004 ^b^ | -0.006 ^b^ |

*Note. N =* 6,330 (one randomly selected twin per pair). CFI = comparative fit index. RMSEA = root mean square error of approximation. SRMR = standardized root mean square residual. Δ denotes change value. ^a^ The change in CFI value from the scalar model to the strict model exceeded the acceptable limit (of 0.010). Consultation of the modification indices and subsequent free estimation of the item 2 parameters provided acceptable deterioration in model fit. ^b^ Change values compared to scalar invariance model. The measurement model was a 5-factor model (age 16: χ^2^ (12) = 31.48, *p <* 0.001, CFI = 0.99, RMSEA = 0.03 [0.02, 0.04], SRMR = 0.01; age 17: χ^2^ (12) = 8.40, *p* = 0.75, CFI = 1.00, RMSEA = 0.00 [0.00, 0.03], SRMR = 0.01; age 22: χ^2^ (12) = 110.13, *p* <.001, CFI = 0.99, RMSEA = 0.06 [0.05, 0.07], SRMR = 0.02). Confirmatory factor analysis results available in full from corresponding author.

**Supplementary Table 29.** Latent growth curve model fit results for results for negative symptoms

|  | Parameters | Log-likelihood | AIC | BIC | χ^2^ value (*df*) | CFI | RMSEA [90% CI] | SRMR |
| --- | --- | --- | --- | --- | --- | --- | --- | --- |
| Intercept only model  **Linear growth model** ^a^  Quadratic growth model ^b^ | 5  **8**  7 | -59574.888  **-59493.463**  -59538.377 | 119159.775  **119002.926**  119090.754 | 119197.007  **119062.497**  119142.879 | 83.728 (4), *p* <.001  **8.211 (1), *p* <.001**  28.503 (2), *p* <.001 | 0.965**0.998**  0.980 | 0.057 [0.047, 0.068]  **0.024 [0.011, 0.041]**  0.060 [0.042, 0.081] | 0.033  **0.014**  0.045 |

*Note. N* = 12,662. Related and unrelated individuals included, using cluster-robust SE. AIC = Akaike’s Information Criterion. BIC = Bayesian Information Criterion. χ^2^ = chi-square value. CFI = comparative fit index. RMSEA = root mean square error of approximation. SRMR = standardized root mean square residual. ^a^ Residual variances were freely estimated at each time-point. ^b^ Quadratic slope variance-covariance parameters not estimated, and residual variances constrained to equality (to achieve identification and over-identification, respectively). A proper solution could not be obtained for an over-identified latent basis model (with residual variances constrained to equality), so no results are reported. Bold typeset indicates model that was selected for subsequent growth mixture modelling.

**Supplementary Table 30.** Parameter estimates from linear growth curve model of negative symptoms

|  | Mean | | |  |  |  | Variance |  |  |
| --- | --- | --- | --- | --- | --- | --- | --- | --- | --- |
|  | Estimate | SE | *z* | *p* |  | Estimate | SE | *z* | *p* |
| Intercept  Slope  16 years  17 years  22 years | 2.26  0.07  -  -  - | 0.04  0.01  -  -  - | 59.45  10.36  -  -  - | <.001  <.001  -  -  - |  | 6.92  0.06  3.44  5.70  5.18 | 0.36  0.06  0.32  0.55  1.80 | 19.18  1.00  10.87  10.33  2.89 | <.001  .32  -  <.001  .004 |
|  | Estimate | SE | *z* | *p* | Standardised  estimate |  |  |  |  |
| Factor covariance | -0.08 | 0.05 | -1.58 | .11 | -0.14 |  |  |  |  |

*Note. N* = 12,662. Related and unrelated individuals included, using cluster-robust SE. Unstandardised estimates (unless otherwise indicated).

**Supplementary Table 31.** Growth mixture model fit results for negative symptoms

| *k* | Model | Par. | Constraints | LL | BIC | AIC | Entropy ^1^ | |
| --- | --- | --- | --- | --- | --- | --- | --- | --- |
| 1  1  2  2  3  3  3  3  3  3  3  3 | Model LCGA  Model 0  Model LCGA  Model 0  Model LCGA  Model 0  Model 1A  Model 1B  Model 1C  Model 2A  Model 2B  Model 2C | 5  8  11  17  17  26  20  20  22  18  15  16 | No growth factor variances  None  No growth factor variances  None  No growth factor variances  None  Within-class residual variances  Between-class residual variances  Between-class growth factor variances  Within-class and between-class residual variances  Within-class residual variances and between-class growth factor variances  Between-class residual variances and between-class growth factor variances | -61820.215  -59459.934  -51529.472  -50727.482  -  -  -  -  -  -53174.429  -  -54517.112 | 123687.658  **118995.433**  103162.846  **101615.540**  -  -  -  -  -  **106518.879**  -  109185.352 | 123650.430  118935.868  103080.945  101488.965  -  -  -  -  -  106384.859  -  109066.223 | -  -  0.790  0.788  -  -  -  -  -  0.708  -  0.684 |  |

*Note. k* = number of classes. Par. = number of estimated parameters (for final model if converged, for unadjusted model if not converged). LL = loglikelihood value. AIC = Akaike’s Information Criterion. BIC = Bayesian Information Criterion. ^1^ = No calculation for 1-class model. Bold typeset indicates lowest BIC value for each *k*-class model. Loglikelihood values replicated for best-fitting *k*-class models using the two random seed values with the highest loglikelihoods.

**Supplementary Table 32.** Parameter estimates for each best fitting *k-*class model for negative symptoms

| *k* | Model | Parameter | Class 1  Mean (*SE*) | *P* (*SE*) | Variance (*SE*) | *P* | Class 2  Mean (*SE*) | *P* | Variance (*SE*) | *P* | Class 3  Mean (*SE*) | *P* | Variance (*SE*) | *P* |
| --- | --- | --- | --- | --- | --- | --- | --- | --- | --- | --- | --- | --- | --- | --- |
| 1 | Model 0 | Intercept  Linear slope  W1  W2  W3  Covariance | 2.256 (0.039)  0.064 (0.007)  -  -  -  - | <.001  <.001  -  -  -  - | 6.903 (0.338)  0.010 (0.027)  3.551 (0.265)  5.622 (0.551)  6.682 (0.943)  -0.069 (0.045) | <.001  .705  <.001<.001<.001  .126 | -  -  -  -  -  - | -  -  -  -  -  - | -  -  -  -  -  - | -  -  -  -  -  - | -  -  -  -  -  - | -  -  -  -  -  - | -  -  -  -  -  - | -  -  -  -  -  - |
| 2 | Model 0 | Intercept  Linear slope  W1  W2  W3  Covariance | 3.682 (0.085)  0.095 (0.011)  -  -  -  - | <.001  <.001  -  -  -  - | 7.089 (0.465)  0.093 (0.041)  5.595 (0.418)  8.220 (0.785)  8.692 (1.387)  -0.269 (0.069) | <.001  .022  <.001<.001<.001  <.001 | 0.189 (0.014)  0.034 (0.008)  -  -  -  - | <.001  <.001  -  -  -  - | 0.031 (0.011)  0.001 (0.001)  0.130 (0.010)  0.142 (0.013)  0.356 (0.058)  0.000 (0.002) | .004  .577  <.001<.001<.001  .819 | -  -  -  -  -  - | -  -  -  -  -  - | -  -  -  -  -  - | -  -  -  -  -  - |
| 3 | Model 2A | Intercept  Linear slope  W1  W2  W3  Covariance | 3.557 (0.150)  0.094 (0.031)  -  -  -  - | <.001  .003  -  -  -  - | 3.525 (0.523)  0.284 (0.044)  1.987 (0.107)  1.987 (0.107)  1.987 (0.107)  -0.868 (0.110) | <.001  <.001  <.001<.001<.001<.001 | 0.779 (0.048)  0.021(0.026)  -  -  -  - | <.001  .422  -  -  -  - | 0.003 (0.240)  0.000 (0.032)  1.987 (0.107)  1.987 (0.107)  1.987 (0.107)  0.000 (0.055) | .991  .997  <.001<.001<.001  .996 | 7.974 (0.219)  0.288 (0.056)  -  -  -  - | <.001  <.001  -  -  -  - | 20.865 (1.532)  1.436 (0.100)  1.987 (0.107)  1.987 (0.107)  1.987 (0.107)  -3.766 (0.293) | <.001  <.001  <.001<.001<.001  <.001 |

*Note. k* = number of classes. W1-W3 = data collection waves 1-3. Variance of W1-W3 represents residual variance at data collection waves 1-3. Model 0: Unconstrained model. Model 2A: Model with within-class and between-class residual variances constrained.

**Supplementary Table 33.** Most likely class classification values for each best fitting *k-*class model for negative symptoms

| *k* | Model |  | Classification probabilities | | | Final class counts and proportions |
| --- | --- | --- | --- | --- | --- | --- |
|  |  |  | Class 1 | Class 2 | Class 3 |  |
| 1 | Model 0 | Class 1 | 1.000 | - | - | 12652 (100%) |
| 2 | Model 0 | Class 1  Class 2 | 0.922  0.028 | 0.078  0.972 | -  - | 6967 (55.07%)  5685 (44.93%) |
| 3 | Model 2A | Class 1  Class 2  Class 3 | 0.727  0.033  0.244 | 0.239  0.967  0.053 | 0.033  0.000  0.704 | 2836 (22.42%)  8745 (69.12%)  1071 (8.47%) |

*Note. k* = number of classes. Model 0: Unconstrained model. Model 2A: Model with within-class and between-class residual variances constrained. Values based on most likely latent class membership

**Supplementary Table 34**. Multinomial logistic regression results for negative symptoms latent trajectory class regressed on GPSs for all GPS *f*

|  | Beta | | Odds Ratio | | |
| --- | --- | --- | --- | --- | --- |
|  | *b* (SE) | Z (P value) | OR (SE) | 95% CI lower bound | 95% CI upper bound |
| EA3_1 |  |  |  |  |  |
| Low-increasing vs mid-increasing | -0.258 (0.029) | **-9.025 (<.001)** | 0.772 (0.022) | 0.739 | 0.817 |
|  |  |  |  |  |  |
| EA3_0.3 |  |  |  |  |  |
| Low-increasing vs mid-increasing | -0.205 (0.028) | -7.226 (<.001) | 0.815 (0.023) | 0.771 | 0.861 |
|  |  |  |  |  |  |
| EA3_0.01 |  |  |  |  |  |
| Low-increasing vs mid-increasing | -0.101 (0.028) | -3.648 (<.001) | 0.904 (0.025) | 0.856 | 0.954 |
|  |  |  |  |  |  |
| IQ_1 |  |  |  |  |  |
| Low-increasing vs mid-increasing | -0.096 (0.028) | **-3.437 (.001)** | 0.908 (0.025) | 0.860 | 0.960 |
|  |  |  |  |  |  |
| IQ_0.3 |  |  |  |  |  |
| Low-increasing vs mid-increasing | -0.086 (0.028) | -3.083 (.002) | 0.917 (0.026) | 0.868 | 0.969 |
|  |  |  |  |  |  |
| IQ_0.01 |  |  |  |  |  |
| Low-increasing vs mid-increasing | -0.017 (0.028) | -0.6177 (.537) | 0.983 (0.028) | 0.930 | 1.038 |
|  |  |  |  |  |  |
| PSYCH_1 |  |  |  |  |  |
| Low-increasing vs mid-increasing | 0.067 (0.028) | 2.425 (.015) | 1.070 (0.030) | 1.013 | 1.130 |
|  |  |  |  |  |  |
| PSYCH_0.3 |  |  |  |  |  |
| Low-increasing vs mid-increasing | 0.068 (0.028) | **2.455 (.014)** | 1.071 (0.030) | 1.014 | 1.131 |
|  |  |  |  |  |  |
| PSYCH_0.01 |  |  |  |  |  |
| Low-increasing vs mid-increasing | 0.040 (0.028) | 1.432 (.152) | 1.040 (0.029) | 0.985 | 1.098 |
|  |  |  |  |  |  |
| GP_1 |  |  |  |  |  |
| Low-increasing vs mid-increasing | 0.094 (0.028) | **3.330 (.001)** | 1.099 (0.031) | 1.039 | 1.161 |
|  |  |  |  |  |  |
| GP_0.3 |  |  |  |  |  |
| Low-increasing vs mid-increasing | 0.093 (0.028) | 3.313 (.001) | 1.098 (0.031) | 1.039 | 1.160 |
|  |  |  |  |  |  |
| GP_0.01 |  |  |  |  |  |
| Low-increasing vs mid-increasing | 0.061 (0.028) | 2.170 (.030) | 1.062 (0.030) | 1.006 | 1.122 |
|  |  |  |  |  |  |
| SCZ_1 |  |  |  |  |  |
| Low-increasing vs mid-increasing | 0.021 (0.028) | **0.758 (.448)** | 1.021 (0.029) | 0.967 | 1.079 |
|  |  |  |  |  |  |
| SCZ_0.3 |  |  |  |  |  |
| Low-increasing vs mid-increasing | 0.020 (0.028) | 0.707 (.480) | 1.020 (0.029) | 0.965 | 1.078 |
|  |  |  |  |  |  |
| SCZ_0.01 |  |  |  |  |  |
| Low-increasing vs mid-increasing | -0.011 (0.028) | -0.407 (.684) | 0.989 (0.027) | 0.937 | 1.044 |
|  |  |  |  |  |  |
| OCD_1 |  |  |  |  |  |
| Low-increasing vs mid-increasing | 0.032 (0.028) | 1.144 (.253) | 1.033 (0.029) | 0.977 | 1.091 |
|  |  |  |  |  |  |
| OCD_0.3 |  |  |  |  |  |
| Low-increasing vs mid-increasing | 0.032 (0.028) | 1.151 (.250) | 1.033 (0.029) | 0.978 | 1.091 |
|  |  |  |  |  |  |
| OCD_0.01 |  |  |  |  |  |
| Low-increasing vs mid-increasing | 0.034 (0.028) | **1.195 (.232)** | 1.034 (0.029) | 0.979 | 1.093 |
|  |  |  |  |  |  |
| MDD_1 |  |  |  |  |  |
| Low-increasing vs mid-increasing | 0.098 (0.028) | **3.461 (.001)** | 1.103 (0.031) | 1.044 | 1.167 |
|  |  |  |  |  |  |
| MDD_0.3 |  |  |  |  |  |
| Low-increasing vs mid-increasing | 0.097 (0.028) | 3.418 (.001) | 1.102 (0.031) | 1.042 | 1.165 |
|  |  |  |  |  |  |
| MDD_0.01 |  |  |  |  |  |
| Low-increasing vs mid-increasing | 0.038 (0.028) | 1.362 (.173) | 1.039 (0.029) | 0.983 | 1.098 |
|  |  |  |  |  |  |
| BIP_1 |  |  |  |  |  |
| Low-increasing vs mid-increasing | -0.058 (0.028) | **-2.073 (.038)** | 0.944 (0.026) | 0.894 | 0.997 |
|  |  |  |  |  |  |
| BIP_0.3 |  |  |  |  |  |
| Low-increasing vs mid-increasing | -0.057 (0.028) | -2.048 (.041) | 0.944 (0.046) | 0.894 | 0.998 |
|  |  |  |  |  |  |
| BIP_0.01 |  |  |  |  |  |
| Low-increasing vs mid-increasing | -0.021 (0.028) | -0.767 (.443) | 0.979 (0.027) | 0.927 | 1.034 |
|  |  |  |  |  |  |
| ASD_1 |  |  |  |  |  |
| Low-increasing vs mid-increasing | 0.036 (0.028) | 1.270 (.204) | 1.036 (0.029) | 0.981 | 1.095 |
|  |  |  |  |  |  |
| ASD_0.3 |  |  |  |  |  |
| Low-increasing vs mid-increasing | 0.036 (0.028) | 1.290 (.197) | 1.037 (0.029) | 0.981 | 1.096 |
|  |  |  |  |  |  |
| ASD_0.01 |  |  |  |  |  |
| Low-increasing vs mid-increasing | 0.047 (0.028) | **1.679 (.093)** | 1.048 (0.029) | 0.992 | 1.107 |
|  |  |  |  |  |  |
| ANOREX_1 |  |  |  |  |  |
| Low-increasing vs mid-increasing | -0.020 (0.027) | -0.712 (.476) | 0.981 (0.027) | 0.929 | 1.035 |
|  |  |  |  |  |  |
| ANOREX_0.3 |  |  |  |  |  |
| Low-increasing vs mid-increasing | -0.020 (0.027) | **-0.714 (.475)** | 0.981 (0.027) | 0.929 | 1.035 |
|  |  |  |  |  |  |
| ANOREX_0.01 |  |  |  |  |  |
| Low-increasing vs mid-increasing | -0.006 (0.028) | -0.220 (.826) | 0.994 (0.027) | 0.941 | 1.049 |
|  |  |  |  |  |  |
| ADHD_1 |  |  |  |  |  |
| Low-increasing vs mid-increasing | 0.067 (0.028) | 2.356 (.018) | 1.069 (0.030) | 1.011 | 1.130 |
|  |  |  |  |  |  |
| ADHD_0.3 |  |  |  |  |  |
| Low-increasing vs mid-increasing | 0.067 (0.028) | **2.371 (.018)** | 1.070 (0.030) | 1.012 | 1.131 |
|  |  |  |  |  |  |
| ADHD_0.01 |  |  |  |  |  |
| Low-increasing vs mid-increasing | 0.050 (0.028) | 1.803 (.071) | 1.052 (0.029) | 0.996 | 1.111 |

*Note.* *N =*7,439. Related and unrelated individuals included, using cluster-robust SE. The ‘low-increasing’ class was used as the reference category. GPS = genome-wide polygenic score (standardised). *f* = fraction of causal markers (at 1, 0.3, 0.01). *b* = unstandardized regression coefficient. Low-increasing = low-increasing class. Mid-increasing = mid-increasing class. EA3 = years of education. IQ = intelligence. PSYCH = ever visited a psychiatrist for nerves, anxiety, tension, or depression. GP = ever visited a general practitioner for nerves, anxiety, tension, or depression. SCZ = schizophrenia. OCD = obsessive compulsive disorder. MDD = major depressive disorder. BIP = bipolar disorder. ASD = autism spectrum disorder. ANOREX = anorexia. ADHD = attention deficit hyperactivity disorder. Bold typeset represents the highest *z* score for each GPS.

**Supplementary Table 35**. Multinomial logistic regression results for negative symptoms latent trajectory class regressed on GPSs for most predictive *f*

|  | | **Single predictor regressions** | | | | | **Multiple predictor regression** | | | | |
| --- | --- | --- | --- | --- | --- | --- | --- | --- | --- | --- | --- |
| **GPS variable** | ***f*** | **Beta** | | **Odds Ratio** | | | **Beta** | | **Odds Ratio** | | |
|  |  | ***b* (SE)** | **Z (*P* value)** | **OR (SE)** | **95% CI lower** | **95% CI upper** | ***b* (SE)** | **Z (*P* value)** | **OR (SE)** | **95% CI lower** | **95% CI upper** |
| **Years of education** | **1** |  | | | | |  | | | | |
| Low-inc vs mid-inc |  | -0.258 (0.029) | -9.025 (<.001)* | 0.772 (0.022) | 0.739 | 0.817 | -0.256 (0.032) | -7.943 (<.001)* | 0.774 (0.025) | 0.727 | 0.825 |
| **IQ** | **1** |  | | | | |  | | | | |
| Low-inc vs mid-inc |  | -0.096 (0.028) | -3.437 (.001)* | 0.908 (0.025) | 0.860 | 0.960 | 0.019 (0.031) | 0.600 (.548) | 1.019 (0.032) | 0.959 | 1.083 |
| **Visited a psychiatrist** | **0.3** |  | | | | |  | | | | |
| Low-inc vs mid-inc |  | 0.068 (0.028) | 2.455 (.014)* | 1.071 (0.030) | 1.014 | 1.131 | 0.023 (0.035) | 0.646 (.518) | 1.023 (0.036) | 0.955 | 1.095 |
| **Visited a GP** | **1** |  | | | | |  | | | | |
| Low-inc vs mid-inc |  | 0.094 (0.028) | 3.330 (.001)* | 1.099 (0.031) | 1.039 | 1.161 | 0.035 (0.036) | 0.950 (.342) | 1.035 (0.038) | 0.964 | 1.112 |
| **Schizophrenia** | **1** |  | | | | |  | | | | |
| Low-inc vs mid-inc |  | 0.021 (0.028) | 0.758 (.448) | 1.021 (0.029) | 0.967 | 1.079 | - | - | - | - | - |
| **OCD** | **0.01** |  | | | | |  | | | | |
| Low-inc vs mid-inc |  | 0.034 (0.028) | 1.195 (.232) | 1.034 (0.029) | 0.979 | 1.093 | - | - | - | - | - |
| **MDD** | **1** |  | | | | |  | | | | |
| Low-inc vs mid-inc |  | 0.098 (0.028) | 3.461 (.001)* | 1.103 (0.031) | 1.044 | 1.167 | 0.055 (0.031) | 1.767 (.077) | 1.057 (0.033) | 0.994 | 1.124 |
| **Bipolar disorder** | **1** |  | | | | |  | | | | |
| Low-inc vs mid-inc |  | -0.058 (0.028) | -2.073 (.038) | 0.944 (0.026) | 0.894 | 0.997 | - | - | - | - | - |
| **ASD** | **0.01** |  | | | | |  | | | | |
| Low-inc vs mid-inc |  | 0.047 (0.028) | 1.679 (.093) | 1.048 (0.029) | 0.992 | 1.107 | - | - | - | - | - |
| **Anorexia** | **0.3** |  | | | | |  | | | | |
| Low-inc vs mid-inc |  | -0.020 (0.027) | -0.714 (.475) | 0.981 (0.027) | 0.929 | 1.035 | - | - | - | - | - |
| **ADHD** | **0.3** |  | | | | |  | | | | |
| Low-inc vs mid-inc |  | 0.067 (0.028) | 2.371 (.018)* | 1.070 (0.030) | 1.012 | 1.131 | 0.001 (0.030) | 0.028 (.978) | 1.001 (0.030) | 0.944 | 1.061 |

*Note. N =*7,439*.* Related and unrelated individuals included, using cluster-robust SE. The ‘low-increasing’ class was used as the reference category. GPS = genome-wide polygenic score (standardised). *f* = fraction of causal markers. *b* = unstandardized regression coefficient. Low-inc = low-increasing class. Mid-inc = mid-increasing class. IQ = intelligence. Visited a psychiatrist = ever visited a psychiatrist for nerves, anxiety, tension, or depression. Visited a GP = ever visited a general practitioner for nerves, anxiety, tension, or depression. OCD = obsessive compulsive disorder. MDD = major depressive disorder. ASD = autism spectrum disorder. ADHD = attention deficit hyperactivity disorder. * = significant at *q* <.05 (FDR-adjusted *p* <.027 and *p* <.008 for single and multiple predictor regressions, respectively).

**Supplementary Table 36**. Multinomial logistic regression results for negative symptoms latent trajectory class regressed on family background variables

|  |  | **Single predictor regressions** | | | | | **Multiple predictor regression, *N* = 11961** | | | | |
| --- | --- | --- | --- | --- | --- | --- | --- | --- | --- | --- | --- |
| **Phenotypic variable** | ***N* for single predictor regressions** | **Beta** | | **Odds Ratio** | | | **Beta** | | **Odds Ratio** | | |
|  |  | ***b* (SE)** | **Z**  **(*P* value)** | **OR (SE)** | **95% CI lower** | **95% CI upper** | ***b* (SE)** | **Z**  **(*P* value)** | **OR (SE)** | **95% CI lower** | **95% CI upper** |
| **Male sex** |  |  | | | | |  | | | | |
| Low-inc vs mid-inc | 12652 | 0.259 (0.046) | 5.603 (<.001)* | 1.295 (0.060) | 1.183 | 1.418 | 0.285 (0.048) | 5.940 (<.001)* | 1.330 (0.064) | 1.211 | 1.461 |
| **SES** |  |  | | | | |  | | | | |
| Low-inc vs mid-inc | 11961 | -0.232 (0.026) | -9.021 (<.001)* | 0.793 (0.020) | 0.754 | 0.834 | -0.239 (0.026) | -9.184 (<.001)* | 0.788 (0.020) | 0.749 | 0.829 |
| **Family history of schizophrenia** |  |  | | | | |  | | | | |
| Low-inc vs mid-inc | 9737 | 0.231 (0.151) | 1.533 (.125) | 1.260 (0.190) | 0.938 | 1.693 | - | - | - | - | - |
| **Family history of bipolar disorder** |  |  | | | | |  | | | | |
| Low-inc vs mid-inc | 9523 | 0.186 (0.126) | 1.477 (.140) | 1.205 (0.152) | 0.941 | 1.543 | - | - | - | - | - |

*Note.* Related and unrelated individuals included, using cluster-robust SE. The ‘low-increasing’ class was used as the reference category. *b* = unstandardized regression coefficient. Low-inc = low-increasing class. Mid-inc = mid-increasing class. SES = socioeconomic status. * = significant at *q* <.05 (FDR-adjusted *p* <.025 and *p* <.05 for single and multiple predictor regressions, respectively).

**Supplementary Table 37**. Multinomial logistic regression results for negative symptoms latent trajectory class regressed on age 7 variables

|  |  | **Single predictor regressions** | | | | | **Multiple predictor regression, *N* = 7919** | | | | |
| --- | --- | --- | --- | --- | --- | --- | --- | --- | --- | --- | --- |
| **Phenotypic variable** | ***N* for single predictor regressions** | **Beta** | | **Odds Ratio** | | | **Beta** | | **Odds Ratio** | | |
|  |  | ***b* (SE)** | **Z**  **(*P* value)** | **OR (SE)** | **95% CI lower** | **95% CI upper** | ***b* (SE)** | **Z**  **(*P* value)** | **OR (SE)** | **95% CI lower** | **95% CI upper** |
| **Educational attainment** |  |  | | | | |  | | | | |
| Low-inc vs mid-inc | 8172 | -0.224 (0.031) | -7.303 (<.001)* | 0.799 (0.025) | 0.752 | 0.849 | -0.071 (0.033) | -2.125 (.034) | 0.932 (0.031) | 0.873 | 0.995 |
| **Life events** |  |  | | | | |  | | | | |
| Low-inc vs mid-inc | 10235 | 0.058 (0.022) | 2.571 (.010)* | 1.059 (0.024) | 1.014 | 1.107 | 0.018 (0.026) | 0.678 (.498) | 1.018 (0.026) | 0.967 | 1.071 |
| **SDQ** |  |  | | | | |  | | | | |
| Low-inc vs mid-inc | 10231 | 0.119 (0.006) | 18.654 (<.001)* | 1.126 (0.007) | 1.112 | 1.141 | 0.117 (0.007) | 15.749 (<.001)* | 1.124 (0.008) | 1.108 | 1.141 |

*Note.* Related and unrelated individuals included, using cluster-robust SE. The ‘low-increasing’ class was used as the reference category. *b* = unstandardized regression coefficient. Low-inc = low-increasing class. Mid-inc = mid-increasing class. SDQ = Strengths and Difficulties Questionnaire). * = significant at *q* <.05 (FDR-adjusted *p* <.05 and *p* <.017 for single and multiple predictor regressions, respectively).

**References**

Andreasen, N. C. (1982). Negative Symptoms in Schizophrenia: Definition and Reliability. *Archives of General Psychiatry*, *39*(7), 784–788. https://doi.org/10.1001/archpsyc.1982.04290070020005

Angold, A., Costello, E. J., Messer, S. C., & Pickles, A. (1995). Development of a short questionnaire for use in epidemiological studies of depression in children and adolescents. *International Journal of Methods in Psychiatric Research*, *5*(4), 237–249.

Bell, V., Halligan, P. W., & Ellis, H. D. (2006). The Cardiff Anomalous Perceptions Scale (CAPS): A New Validated Measure of Anomalous Perceptual Experience. *Schizophrenia Bulletin*, *32*(2), 366–377. https://doi.org/10.1093/schbul/sbj014

Chen, F. F. (2007). Sensitivity of Goodness of Fit Indexes to Lack of Measurement Invariance. *Structural Equation Modeling: A Multidisciplinary Journal*, *14*(3), 464–504. https://doi.org/10.1080/10705510701301834

Choi, S. W., Mak, T. S.-H., & O’Reilly, P. F. (2020). Tutorial: A guide to performing polygenic risk score analyses. *Nature Protocols*, *15*(9), 2759–2772. https://doi.org/10.1038/s41596-020-0353-1

Coddington, R. D. (1972). The significance of life events as etiologic factors in the diseases of children: II. A study of a normal population. *Journal of Psychosomatic Research*, *16*(3), 205–213. https://doi.org/10.1016/0022-3999(72)90045-1

Demontis, D., Walters, R. K., Martin, J., Mattheisen, M., Als, T. D., Agerbo, E., Baldursson, G., Belliveau, R., Bybjerg-Grauholm, J., Bækvad-Hansen, M., Cerrato, F., Chambert, K., Churchhouse, C., Dumont, A., Eriksson, N., Gandal, M., Goldstein, J. I., Grasby, K. L., Grove, J., … Neale, B. M. (2019). Discovery of the first genome-wide significant risk loci for attention deficit/hyperactivity disorder. *Nature Genetics*, *51*(1), 63–75. https://doi.org/10.1038/s41588-018-0269-7

Duncan, L., Yilmaz, Z., Gaspar, H., Walters, R., Goldstein, J., Anttila, V., Bulik-Sullivan, B., Ripke, S., Thornton, L., Hinney, A., Daly, M., Sullivan, P. F., Zeggini, E., Breen, G., Bulik, C. M., Duncan, L., Yilmaz, Z., Gaspar, H., Walters, R., … Bulik, C. M. (2017). Significant Locus and Metabolic Genetic Correlations Revealed in Genome-Wide Association Study of Anorexia Nervosa. *American Journal of Psychiatry*, *174*(9), 850–858. https://doi.org/10.1176/appi.ajp.2017.16121402

Flora, D. B., & Flake, J. K. (2017). The purpose and practice of exploratory and confirmatory factor analysis in psychological research: Decisions for scale development and validation. *Canadian Journal of Behavioural Science / Revue Canadienne Des Sciences Du Comportement*, *49*(2), 78–88. https://doi.org/10.1037/cbs0000069

Freeman, D., Garety, P. A., Bebbington, P. E., Smith, B., Rollinson, R., Fowler, D., Kuipers, E., Ray, K., & Dunn, G. (2005). Psychological investigation of the structure of paranoia in a non-clinical population. *The British Journal of Psychiatry*, *186*(5), 427–435. https://doi.org/10.1192/bjp.186.5.427

Goodman, R. (1997). The Strengths and Difficulties Questionnaire: A Research Note. *Journal of Child Psychology and Psychiatry*, *38*(5), 581–586. https://doi.org/10.1111/j.1469-7610.1997.tb01545.x

Grimm, K. J., Ram, N., & Hamagami, F. (2011). Nonlinear Growth Curves in Developmental Research. *Child Development*, *82*(5), 1357–1371. https://doi.org/10.1111/j.1467-8624.2011.01630.x

Grove, J., Ripke, S., Als, T. D., Mattheisen, M., Walters, R. K., Won, H., Pallesen, J., Agerbo, E., Andreassen, O. A., Anney, R., Awashti, S., Belliveau, R., Bettella, F., Buxbaum, J. D., Bybjerg-Grauholm, J., Bækvad-Hansen, M., Cerrato, F., Chambert, K., Christensen, J. H., … Børglum, A. D. (2019). Identification of common genetic risk variants for autism spectrum disorder. *Nature Genetics*, *51*(3), 431–444. https://doi.org/10.1038/s41588-019-0344-8

Havers, L., Cardno, A., Freeman, D., & Ronald, A. (2022). The latent structure of negative symptoms in the general population in adolescence and emerging adulthood. *Schizophrenia Bulletin Open*, sgac009. https://doi.org/10.1093/schizbullopen/sgac009

IOCDF-GC and OCGAS. (2018). Revealing the complex genetic architecture of obsessive-compulsive disorder using meta-analysis. *Molecular Psychiatry*, *23*(5), 1181–1188. https://doi.org/10.1038/mp.2017.154

Lee, J. J., Wedow, R., Okbay, A., Kong, E., Maghzian, O., Zacher, M., Nguyen-Viet, T. A., Bowers, P., Sidorenko, J., Karlsson Linnér, R., Fontana, M. A., Kundu, T., Lee, C., Li, H., Li, R., Royer, R., Timshel, P. N., Walters, R. K., Willoughby, E. A., … Cesarini, D. (2018). Gene discovery and polygenic prediction from a genome-wide association study of educational attainment in 1.1 million individuals. *Nature Genetics*, *50*(8), 1112–1121. https://doi.org/10.1038/s41588-018-0147-3

Mackinnon, S., Curtis, R., & O’Connor, R. (2022). A Tutorial in Longitudinal Measurement Invariance and Cross-lagged Panel Models Using Lavaan. *Meta-Psychology*, *6*. https://doi.org/10.15626/MP.2020.2595

Neale Lab. (2017). *Rapid GWAS of thousands of phenotypes for 337,000 samples in the UK Biobank*. Neale Lab. http://www.nealelab.is/blog/2017/7/19/rapid-gwas-of-thousands-of-phenotypes-for-337000-samples-in-the-uk-biobank

Osborne, J., & Fitzpatrick, D. (2019). Replication Analysis in Exploratory Factor Analysis: What it is and why it makes your analysis better. *Practical Assessment, Research, and Evaluation*, *17*(1). https://doi.org/10.7275/h0bd-4d11

Pardiñas, A. F., Holmans, P., Pocklington, A. J., Escott-Price, V., Ripke, S., Carrera, N., Legge, S. E., Bishop, S., Cameron, D., Hamshere, M. L., Han, J., Hubbard, L., Lynham, A., Mantripragada, K., Rees, E., MacCabe, J. H., McCarroll, S. A., Baune, B. T., Breen, G., … Walters, J. T. R. (2018). Common schizophrenia alleles are enriched in mutation-intolerant genes and in regions under strong background selection. *Nature Genetics*, *50*(3), 381–389. https://doi.org/10.1038/s41588-018-0059-2

Psychiatric GWAS Consortium Bipolar Disorder Working Group. (2011). Large-scale genome-wide association analysis of bipolar disorder identifies a new susceptibility locus near ODZ4. *Nature Genetics*, *43*(10), 977–983. https://doi.org/10.1038/ng.943

Purves, K. L., Coleman, J. R. I., Meier, S. M., Rayner, C., Davis, K. A. S., Cheesman, R., Bækvad-Hansen, M., Børglum, A. D., Wan Cho, S., Jürgen Deckert, J., Gaspar, H. A., Bybjerg-Grauholm, J., Hettema, J. M., Hotopf, M., Hougaard, D., Hübel, C., Kan, C., McIntosh, A. M., Mors, O., … Eley, T. C. (2020). A major role for common genetic variation in anxiety disorders. *Molecular Psychiatry*, *25*(12), 3292–3303. https://doi.org/10.1038/s41380-019-0559-1

Putnick, D. L., & Bornstein, M. H. (2016). Measurement Invariance Conventions and Reporting: The State of the Art and Future Directions for Psychological Research. *Developmental Review : DR*, *41*, 71–90. https://doi.org/10.1016/j.dr.2016.06.004

Ronald, A., Sieradzka, D., Cardno, A. G., Haworth, C. M. A., McGuire, P., & Freeman, D. (2014). Characterization of Psychotic Experiences in Adolescence Using the Specific Psychotic Experiences Questionnaire: Findings From a Study of 5000 16-Year-Old Twins. *Schizophrenia Bulletin*, *40*(4), 868–877. https://doi.org/10.1093/schbul/sbt106

Savage, J. E., Jansen, P. R., Stringer, S., Watanabe, K., Bryois, J., de Leeuw, C. A., Nagel, M., Awasthi, S., Barr, P. B., Coleman, J. R. I., Grasby, K. L., Hammerschlag, A. R., Kaminski, J. A., Karlsson, R., Krapohl, E., Lam, M., Nygaard, M., Reynolds, C. A., Trampush, J. W., … Posthuma, D. (2018). Genome-wide association meta-analysis in 269,867 individuals identifies new genetic and functional links to intelligence. *Nature Genetics*, *50*(7), 912–919. https://doi.org/10.1038/s41588-018-0152-6

Selzam, S., McAdams, T. A., Coleman, J. R. I., Carnell, S., O’Reilly, P. F., Plomin, R., & Llewellyn, C. H. (2018). Evidence for gene-environment correlation in child feeding: Links between common genetic variation for BMI in children and parental feeding practices. *PLoS Genetics*, *14*(11). https://doi.org/10.1371/journal.pgen.1007757

Selzam, S., Ritchie, S. J., Pingault, J.-B., Reynolds, C. A., O’Reilly, P. F., & Plomin, R. (2019). Comparing Within- and Between-Family Polygenic Score Prediction. *American Journal of Human Genetics*, *105*(2), 351–363. https://doi.org/10.1016/j.ajhg.2019.06.006

Steinmetz, H. (2013). Analyzing Observed Composite Differences Across Groups. *Methodology*, *9*(1), 1–12. https://doi.org/10.1027/1614-2241/a000049

van de Schoot, R., Lugtig, P., & Hox, J. (2012). A checklist for testing measurement invariance. *European Journal of Developmental Psychology*, *9*(4), 486–492. https://doi.org/10.1080/17405629.2012.686740

Vilhjálmsson, B. J., Yang, J., Finucane, H. K., Gusev, A., Lindström, S., Ripke, S., Genovese, G., Loh, P.-R., Bhatia, G., Do, R., Hayeck, T., Won, H.-H., Ripke, S., Neale, B. M., Corvin, A., Walters, J. T. R., Farh, K.-H., Holmans, P. A., Lee, P., … Price, A. L. (2015). Modeling Linkage Disequilibrium Increases Accuracy of Polygenic Risk Scores. *The American Journal of Human Genetics*, *97*(4), 576–592. https://doi.org/10.1016/j.ajhg.2015.09.001

Widaman, K. F., & Reise, S. P. (1997). Exploring the measurement invariance of psychological instruments: Applications in the substance use domain. In *The science of prevention: Methodological advances from alcohol and substance abuse research* (pp. 281–324). American Psychological Association. https://doi.org/10.1037/10222-009

Wray, N. R., Ripke, S., Mattheisen, M., Trzaskowski, M., Byrne, E. M., Abdellaoui, A., Adams, M. J., Agerbo, E., Air, T. M., Andlauer, T. M. F., Bacanu, S.-A., Bækvad-Hansen, M., Beekman, A. F. T., Bigdeli, T. B., Binder, E. B., Blackwood, D. R. H., Bryois, J., Buttenschøn, H. N., Bybjerg-Grauholm, J., … Sullivan, P. F. (2018). Genome-wide association analyses identify 44 risk variants and refine the genetic architecture of major depression. *Nature Genetics*, *50*(5), 668–681. https://doi.org/10.1038/s41588-018-0090-3

Zammit, S., Owen, M. J., Evans, J., Heron, J., & Lewis, G. (2011). Cannabis, COMT and psychotic experiences. *The British Journal of Psychiatry*, *199*(5), 380–385. https://doi.org/10.1192/bjp.bp.111.091421
